# Supplementary material for: The calcineurin pathway regulates extreme thermotolerance, cell membrane and wall integrity, antifungal resistance, and virulence in Candida auris
Source: PLoS Pathog. 2025 Jul 28;21(7):e1013363. doi: 10.1371/journal.ppat.1013363 (PMC12324677; doi:10.1371/journal.ppat.1013363)
Supplement: S1 Table — (DOCX) [file ppat.1013363.s001.docx]

**S1 Table. Strains used in this study**

| Strain | Genotype | Parent | Reference |
| --- | --- | --- | --- |
| B8441 | Wild-type |  | (1) |
| B11220 | Wild-type |  | (1) |
| B11221 | Wild-type |  | (1) |
| B11245 | Wild-type |  | (1) |
| YSBA4 | *bcy1*∆::*NAT* | B8441 | (2) |
| YSBA24 | *tpk1*∆::*NAT tpk2*∆::*HYG* | YSBA17 | (2) |
| YSBA119 | *sapa3*∆::*NAT* | B8441 | (3) |
| YSBA99 | *cna1*∆::*NAT* | B8441 | This study |
| YSBA102 | *cnb1*∆::*NAT* | B8441 | This study |
| YSBA105 | *crz1*∆::*NAT* | B8441 | This study |
| YSBA110 | *cna1*Δ::*CNA1-HYG* | YSBA99 | This study |
| YSBA111 | *cnb1*Δ::*CNB1-HYG* | YSBA102 | This study |
| YSBA143 | *crz2*∆::*NAT* | B8441 | This study |
| YSBA153 | *crz1*∆::*NAT crz2*∆::*NEO* | YSBA105 | This study |
| YSBA158 | *crz1*Δ::*CRZ1-HYG* | YSBA105 | This study |
| YSBA172 | *cnb1*∆::*NAT cna1*∆::*HYG* | YSBA102 | This study |
| YSBA289 | *crz1*Δ::*CRZ1*-*mCherry*-*NEO* | YSBA105 | This study |
| YSBA313 | *crz1*Δ::*CRZ1*-*mCherry*-*NEO* *cna1*Δ | YSBA289 | This study |
| YSBA332 | *cna1*∆::*NAT* | B11220 | This study |
| YSBA336 | *cna1*∆::*NEO* | B11221 | This study |
| YSBA365 | *cna1*∆::*NEO* | B11245 | This study |
| YSBA366 | *mkc1*∆*::NEO* | B8441 | This study |
| YSBA368 | *cna1*∆::*NAT mkc1*∆::*NEO* | YSBA99 | This study |

**References**

1. Burrack LS, Todd RT, Soisangwan N, Wiederhold NP, Selmecki A. Genomic diversity across *Candida auris* clinical isolates shapes rapid development of antifungal resistance *in vitro* and *in vivo*. mBio. 2022;13(4):e0084222.

2. Kim JS, Lee KT, Lee MH, Cheong E, Bahn YS. Adenylyl cyclase and protein kinase A play redundant and distinct roles in growth, differentiation, antifungal drug resistance, and pathogenicity of *Candida auris*. mBio. 2021;12(5):e0272921.

3. Kim JS, Lee KT, Bahn YS. Deciphering the regulatory mechanisms of the cAMP/protein kinase A pathway and their roles in the pathogenicity of *Candida auris*. Microbiol Spectr. 2023;11(5):e0215223.
