# Supplementary material for: The calcineurin pathway regulates extreme thermotolerance, cell membrane and wall integrity, antifungal resistance, and virulence in Candida auris
Source: PLoS Pathog. 2025 Jul 28;21(7):e1013363. doi: 10.1371/journal.ppat.1013363 (PMC12324677; doi:10.1371/journal.ppat.1013363)
Supplement: S2 Table — (DOCX) [file ppat.1013363.s002.docx]

**S2 Table. Primers used in this study**

| Name | Primer description | Sequence (5’ to 3’) |
| --- | --- | --- |
| B11103 | pV1025 forward-extended primer | CTAGAACTAGTGGATCTGAA |
| B11104 | pV1025 reverse-extended primer | TAAGAGTGAAATTCTGGAAA |
| B11105 | NAT split primer 1 | CCATTGACTAAGGTTTTCCC |
| B11106 | NAT split primer 2 | TTCAGTAGCCAAACCCATC |
| B11107 | pV1025 Diagnostic screening primer 1 | TCAGTGGCAAATCCTAACC |
| B11108 | pV1025 Diagnostic screening primer 1 | AGAGAAAATACCCGTGACG |
| B12462 | pYM70 forward-extended primer 1 | CACATTTCCCCGAAAAGTGC |
| B12463 | pYM70 reverse-extended primer 2 | TAACTTGCACTACCTCATCG |
| B12464 | HYG split primer 1 | TGCTGATTTGTCTCAAACTT |
| B12465 | HYG split primer 2 | TACCATTATCAGTCAAAACA |
| B12486 | pYM70 Diagnostic screening primer 1 | TGCGGCACAATTGAATAGGG |
| B12487 | pYM70 Diagnostic screening primer 1 | CGGTGATGACGGTGAAAACC |
| B16376 | pTO149 forward-extended primer 1 | GACATGGAGGCCCAGAATAC |
| B16377 | pTO149 reverse-extended primer 2 | CAGTATAGCGACCAGCATTC |
| B16378 | NEO split primer 1 | TGCTCCTGCCGAGAAAGTAT |
| B16379 | NEO split primer 2 | GCTCTTCGTCGAGATCATCC |
| B16380 | pTO149 Diagnostic screening primer 1 | ACATGGGGATGTATGGGCTA |
| B16381 | pTO149 Diagnostic screening primer 1 | TTTTCGCCTCGACATCATCT |
| B14328 | *CNA1* 5’-flanking region primer L1 | TGTTGAAACACAAGGGCAAA |
| B13428 | *CNA1* 5’-flanking region primer L2 | TTCAGATCCACTAGTTCTAGTTGTTATCGACGGGGGACAT |
| B13429 | *CNA1* 3’-flanking region primer R1 | TTTCCAGAATTTCACTCTTATGAGAAAACTCTCTGGATAA |
| B14329 | *CNA1* 3’-flanking region primer R2 | CCTCCCTTGGTCTTCTCACA |
| B13431 | *CNA1* 5’-screening primer SO | GAACCTGGAAGATTCATGGT |
| B13432 | *CNA1* 3’-sreening primer SO2 | TTTTCCTCTCAAAGCTTGCA |
| B13433 | *CNA1* Southern blot probe primer PO | GCCTTCCTCTGTTACATAAA |
| B13434 | *CNB1* 5’-flanking region primer L1 | GCTAGTCAAGAATGGCATCA |
| B13435 | *CNB1* 5’-flanking region primer L2 | CAGATCCACTAGTTCTAGGTGGATGATGCTAACCCCAT |
| B13436 | *CNB1* 3’-flanking region primer R1 | TCCAGAATTTCACTCTTATGACTTTGAACAATATTTAA |
| B13437 | *CNB1* 3’-flanking region primer R2 | AGCAGTGTGCTTTCTTTACC |
| B13438 | *CNB1* 5’-screening primer SO | CGACAGTGAACTCCTCGAAT |
| B13439 | *CNB1* 3’-sreening primer SO2 | GACACCAAAGCTGTGATCAT |
| B13440 | *CNB1* Southern blot probe primer PO | GGCCCCACTCCCATCGCTAT |
| B13498 | *CRZ1* 5’-flanking region primer L1 | CCGGAGAGAAAATTGGATTT |
| B13499 | *CRZ1* 5’-flanking region primer L2 | TTCAGATCCACTAGTTCTAGTTCACGACGTTCAGAGACAT |
| B13500 | *CRZ1* 3’-flanking region primer R1 | TTTCCAGAATTTCACTCTTACCATCTCGCCACCAGACTAG |
| B13501 | *CRZ1* 3’-flanking region primer R2 | TCTTGTATTGATGAAAGCCA |
| B13502 | *CRZ1* 5’-screening primer SO | GTGCATGAAAACACAGATCA |
| B13503 | *CRZ1* 3’-sreening primer SO2 | TCTCCAATGCGATGTCGAAG |
| B13504 | *CRZ1* Southern blot probe primer PO | GTCTTTGGAGAGGACCGAGG |
| B16963 | *CNA1* Internal screening primer LP | AGCTATTGAGCCAGGAACCA |
| B16964 | *CNA1* Internal screening primer RP | GGAGGGACCACGTAAAGACA |
| B16574 | *CNB1* Internal screening primer LP | CCACGCTTCTAGAGTCCTTCA |
| B16575 | *CNB1* Internal screening primer RP | TGTTCAAAGTCAACGCAGATG |
| B16576 | *CRZ1* Internal screening primer LP | TCCCAACTCGTCATCCTACC |
| B16577 | *CRZ1* Internal screening primer RP | CTGGCCAGGAGAATTCTGAG |
| B16989 | CLP for *CNA1* complementation | CGATGGCGTGTATGGCAACA |
| B16990 | CRP for *CNA1* complementation | GAAAGATGCGGTAGACCATA |
| B16837 | CLP for *CNB1* complementation | GGTCTTTATGCTGCTGAAGG |
| B16838 | CRP for *CNB1* complementation | AGTGGCTGAGAGGCAATTTG |
| B16907 | CLP for *CRZ1* complementation | ATGAAAGCCGTTGTCATTGA |
| B16908 | CRP for *CRZ1* complementation | CCAATGACATAACTTTTCCA |
| B17049 | *CNA1* 5’-flanking region primer L1 (HYG) | GCGTGTATGGCAACATGAAC |
| B17050 | CNA1 5’-flanking region primer L2 (HYG) | GCACTTTTCGGGGAAATGTGGCAAGAGGTATTTGAACGGG |
| B17051 | CNA1 3’-flanking region primer R1 (HYG) | CGATGAGGTAGTGCAAGTTAGTACACCCAATCTAGGATCT |
| B17052 | CNA1 3’-flanking region primer R2 (HYG) | CGGACTCGTAGAATCGAAGC |
| B17053 | CNA1 5’-screening primer SO (HYG) | CTGCCATTGACGAATTGTTG |
| B17055 | CNA1 3’-sreening primer SO2 (HYG) | CAAGAACACCTGCTCATCCA |
| B17054 | CNA1 Southern blot probe primer PO (HYG) | TCATTTTGAAGGCCTAGTGTCA |
| B17100 | *CNA1* sequencing primer 1 | ATGTCTGTGAGAAAAAAGTT |
| B17101 | *CNA1* sequencing primer 2 | CCACCAGGCTTCGAGAATTA |
| B17102 | *CNA1* sequencing primer 3 | TCGAATGTACAAGCGCACGA |
| B17326 | Screening primer for *CNA1* complementation | CTCTCGTGCCGTATTTGACA |
| B21391 | *CNA1* 5’-flanking region primer L1  (B11220, B11221, B11245) | GGCCACGGTGAAGTATGTTT |
| B21392 | *CNA1* 5’-flanking region primer L2  (B11220, B11221) | TTCAGATCCACTAGTTCTAGGCAAGAGGTATTTGAACGGG |
| B21393 | *CNA1* 3’-flanking region primer R1  (B11220) | TTTCCAGAATTTCACTCTTATTACACCCGATCTAGGATCT |
| B21394 | *CNA1* 3’-flanking region primer R2  (B11220, B11221) | CCCAGAAAGATGCGGTAGAC |
| B17783 | *CNA1* 3’-flanking region primer L2 (NEO)  (B11221) | gtattctgggcctccatgtcGCAAGAGGTATTTGAACGGG |
| B17784 | *CNA1* 3’-flanking region primer R1 (NEO)  (B11221) | gaatgctggtcgctatactgGTACACCCAATCTAGGATCT |
| B21398 | *CNA1* 3’-flanking region primer R2  (B11245) | TCGTGATAGCAAATCGCAGA |
| B21476 | *CNA1* Internal screening primer LP (B11245) | TGCAAGCACTTGACCGATTA |
| B21477 | *CNA1* Internal screening primer RP(B11245) | GGGAGGACCACGTAAAGACA |
| B21798 | *CNA1* 5’-flanking region primer L2 (NEO)  (B11245) | gtattctgggcctccatgtcGCAAGAGGTATTTGAACAGG |
| B21799 | *CNA1* 3’-flanking region primer R1 (NEO)  (B11245) | gaatgctggtcgctatactgGAAAATCACCGACATGTTGG |
| B13364 | *CNA1* qRT-PCR primer 1 | GAATGTACAAGCGCACGAAA |
| B13365 | *CNA1* qRT-PCR primer 2 | GCACTGCAGCCTTGTTGTTA |
| B13366 | *CNB1* qRT-PCR primer 1 | GAGTGGGGCCATTGATAAGA |
| B13367 | *CNB1* qRT-PCR primer 2 | ATCGTACCGTCGTGATCCTC |
| B17348 | Screening primer for *CNB1* complementation | GAGCCGACAGTGAACTCCTC |
| B17349 | Screening primer for *CRZ1* complementation | CTACCGACTCTGAGGCAAGG |
| B16985 | *CNB1* sequencing primer 1 | AACCCAAGAAAGCGTTTGGG |
| B16986 | *CNB1* sequencing primer 2 | ATATGATATCGACAGAGATG |
| B16991 | *CRZ1* sequencing primer 1 | ATATCTTTCTACGCTTGCAG |
| B16992 | *CRZ1* sequencing primer 2 | ATCCTAACCCTTCTGGGCTC |
| B16993 | *CRZ1* sequencing primer 3 | TCCGCAAGTAACAGCTTGAA |
| B13492 | *CRZ1* qRT-PCR primer 1 | GTGTCGGTGAAAATGCTGTG |
| B13493 | *CRZ1* qRT-PCR primer 2 | ATGGCAGGCATACAAAGAGG |
| B17333 | *CRZ2* 5’-flanking region primer L1 | TGGGGGTTTCTTGAAAAGTG |
| B17334 | *CRZ2* 5’-flanking region primer L2 | TTCAGATCCACTAGTTCTAGGGGAGGAATTTGGGTGGATG |
| B17335 | *CRZ2* 3’-flanking region primer R1 | TTTCCAGAATTTCACTCTTAACACCGTGAGGTGACACCGG |
| B17336 | *CRZ2* 3’-flanking region primer R2 | TAGACGGCTGAAGGCAAACT |
| B17337 | *CRZ2* 5’-screening primer SO | CGTCTTTTGTCAGCCGTGTA |
| B17339 | *CRZ2* 3’-sreening primer SO2 | AGGTTTGCCTGACCTTGTTG |
| B17338 | *CRZ2* Southern blot probe primer PO | TGGTGAGCTACCGAAAAACC |
| B17340 | *CRZ2* Internal screening primer LP | TCAGCAAGTGCAACAAGTCC |
| B17341 | *CRZ2* Internal screening primer RP | CGTCCAGGTCTCTTCTTTGC |
| B17755 | *CRZ2* 5’-flanking region primer L1 (NEO) | TGGGGGTTTCTTGAAAAGTG |
| B17756 | *CRZ2* 5’-flanking region primer L2 (NEO) | gtattctgggcctccatgtcGGGAGGAATTTGGGTGGATG |
| B17757 | *CRZ2* 3’-flanking region primer R1 (NEO) | gaatgctggtcgctatactgACACCGTGAGGTGACACCGG |
| B17758 | *CRZ2* 3’-flanking region primer R2 (NEO) | TAGACGGCTGAAGGCAAACT |
| B17759 | *CRZ2* 5’-screening primer SO (NEO) | CGTCTTTTGTCAGCCGTGTA |
| B17761 | *CRZ2* 3’-sreening primer SO2 (NEO) | AGGTTTGCCTGACCTTGTTG |
| B17760 | *CRZ2* Southern blot probe primer PO (NEO) | CCATCCTTTCCCTGATTTGA |
| B20346 | *CRZ2* qRT-PCR primer 1 | TCAGCAAGTGCAACAAGTCC |
| B20347 | *CRZ2* qRT-PCR primer 2 | CTGACTGCGTCCTTGGAAAT |
| B11749 | *ACT1* qRT-PCR primer 1 | TTGCTCCTGAAGAACACCCT |
| B11750 | *ACT1* qRT-PCR primer 2 | GCAGGAACGTTGAAGGTCTC |
| B12554 | *ERG11* qRT-PCR primer 1 | TGCCCATCGTCTACAACCTT |
| B12555 | *ERG11* qRT-PCR primer 2 | TCTCTCTGCACAGCTCGAAA |
| B12909 | *ERG6* qRT-PCR primer 1 | AGAGACCAAGAGTTCGCCAA |
| B12910 | *ERG6* qRT-PCR primer 2 | TTAGCAACGTCAGCAGCATC |
| B12660 | *FKS1* qRT-PCR primer 1 | CGAAGAACACGGTCAGGACA |
| B12661 | *FKS1* qRT-PCR primer 2 | CCTCAGGGGTCAAGACGTTC |
| B17569 | *FKS2* qRT-PCR primer 1 | AACTCCGATGACGTTGAACC |
| B17570 | *FKS2* qRT-PCR primer 2 | TTGAGCCTCGGAGTTGTCTT |
| B13289 | *CHS1* qRT-PCR primer 1 | GCCTGAAAGTATCCCGGAGT |
| B13290 | *CHS1* qRT-PCR primer 2 | CCAAATCCTAGTCGCATGCC |
| B13291 | *CHS2* qRT-PCR primer 1 | CGGCAGAACAGTTTACGACC |
| B13292 | *CHS2* qRT-PCR primer 2 | GGGCTTCTGTCTCACCTCTT |
| B13293 | *CHS3* qRT-PCR primer 1 | GGAGAGAGAAGATGGGGCTC |
| B13294 | *CHS3* qRT-PCR primer 2 | GTGGTATTGTGGCAAACGGT |
| B13295 | *CHS4* qRT-PCR primer 1 | GGGTGAAGTTGTCGAATCGG |
| B13296 | *CHS4* qRT-PCR primer 2 | GGCACAGATGGAGAGCATTG |
| B13297 | *CHS5* qRT-PCR primer 1 | GTGGGTAAACTCGATGCGTC |
| B13298 | *CHS5* qRT-PCR primer 2 | TAACAATCGAGCCGGCTTTG |
| B13299 | *CHS6* qRT-PCR primer 1 | TGCACACATACATTGGCGAG |
| B13300 | *CHS6* qRT-PCR primer 2 | CTGGATCGTCTGCACACATG |
| B13301 | *CHS7* qRT-PCR primer 1 | GGTATTGTGGGTGCCTTGTG |
| B13302 | *CHS7* qRT-PCR primer 2 | ATACCCACATCGACCGAGTC |
| B13305 | *CDA2* qRT-PCR primer 1 | CAGCTCCAGTGGTCGATTTG |
| B13306 | *CDA2* qRT-PCR primer 2 | TTGCACGAACTCTGTTGTCG |
| B20922 | CLP for *CRZ1-mCherry* complementation | GGGCCCATCCATGGTCCATTGAGGGT |
| B19089 | CRP for *CRZ1-mCherry* complementation | GCGGCCGCGTCTGGTGGCGAGATGGCGA |
| B20650 | *MKC1* 5’-flanking region primer L1 | CCTCGAGGTCGGACACTACT |
| B20651 | *MKC1* 5’-flanking region primer L2 | GTATTCTGGGCCTCCATGTCGGTTGAGATATAGGAGTTTC |
| B20652 | *MKC1* 3’-flanking region primer R1 | GTGTAGAATGGGTATGGAGTACAGCATCAAATCCAGCTCC |
| B20653 | *MKC1* 3’-flanking region primer R2 | TTACCAAGGATGGCAGGAAG |
| B20654 | *MKC1* Internal screening primer LP | TGACCGACTCGCACTATCAG |
| B20655 | *MKC1* Internal screening primer RP | GTCCACGGTGATTCTCTGGT |
