# Supplementary figures and images for: The calcineurin pathway regulates extreme thermotolerance, cell membrane and wall integrity, antifungal resistance, and virulence in Candida auris
Source: PLoS Pathog. 2025 Jul 28;21(7):e1013363. doi: 10.1371/journal.ppat.1013363 (PMC12324677; doi:10.1371/journal.ppat.1013363)

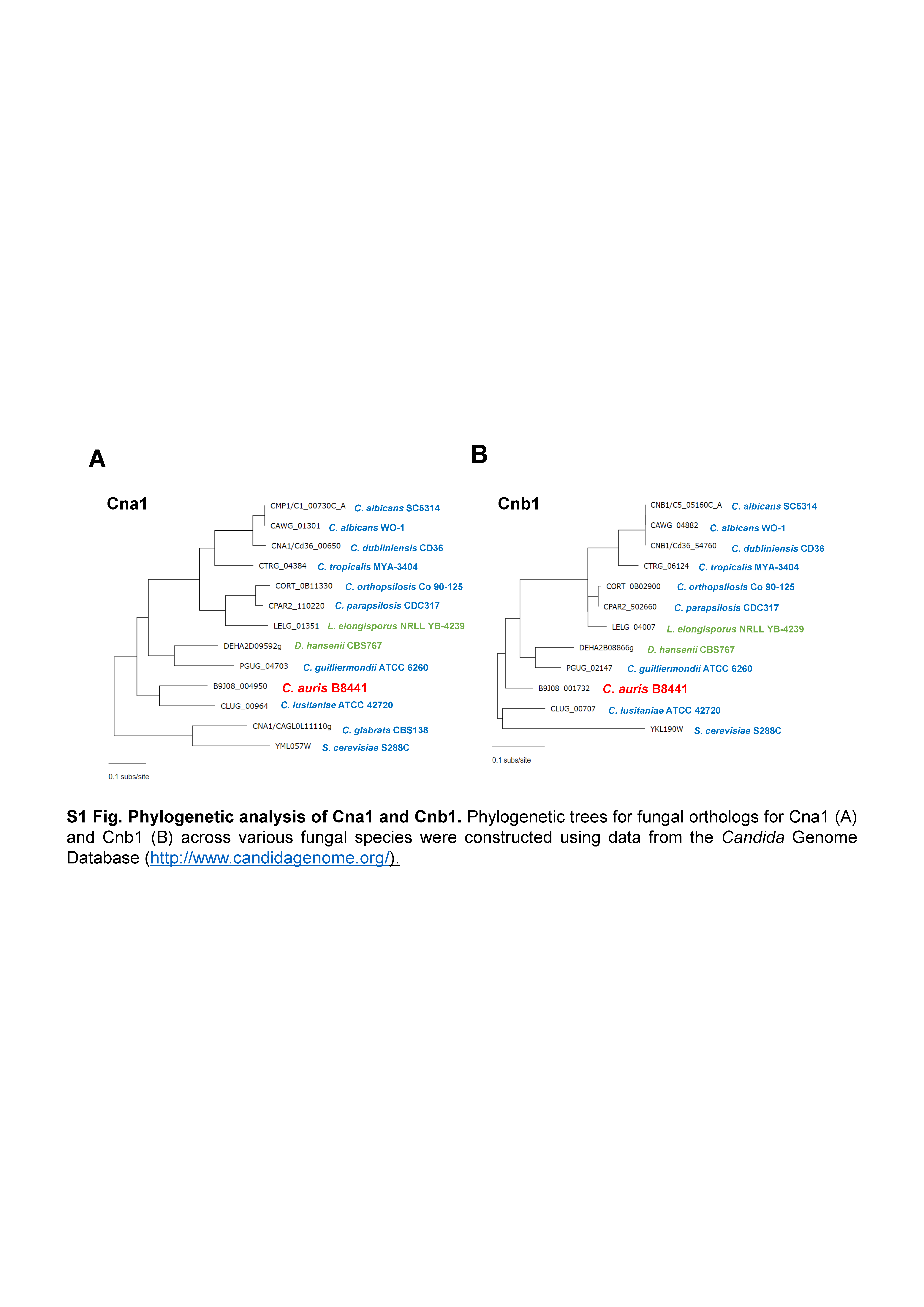

Supplement: S1 Fig — Phylogenetic trees for fungal orthologs for Cna1 (A) and Cnb1 (B) across various fungal species were constructed using data from the Candida Genome Database (http://www.candidagenome.org/). (TIF) [file ppat.1013363.s003.tif]

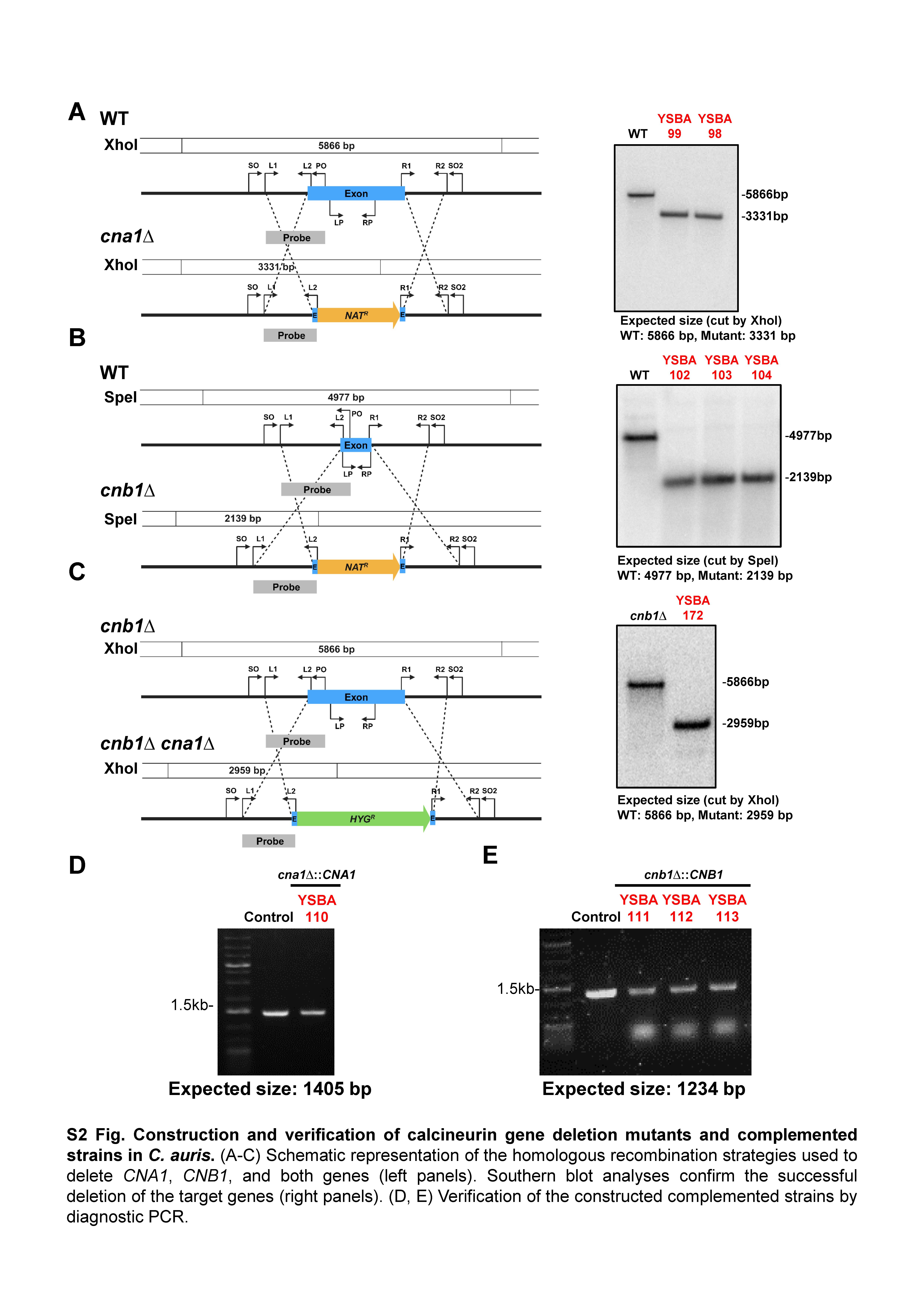

Supplement: S2 Fig — (A-C) Schematic representation of the homologous recombination strategies used to delete CNA1, CNB1, and both genes (left panels). Southern blot analyses confirm the successful deletion of the target genes (right panels). (D, E) Verification of the constructed complemented strains by diagnostic PCR. (TIF) [file ppat.1013363.s004.tif]

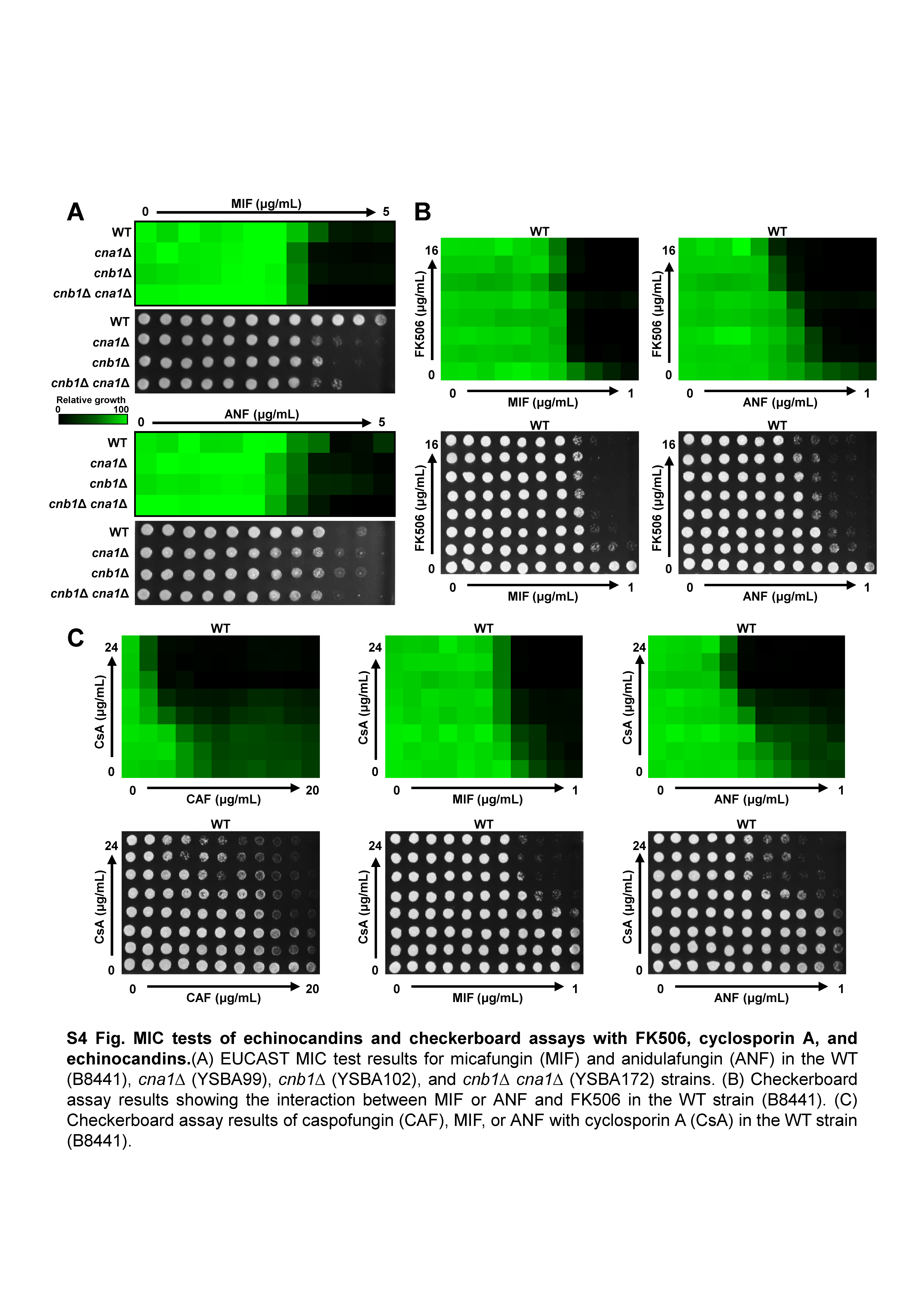

Supplement: S4 Fig — (A) EUCAST MIC test results for micafungin (MIF) and anidulafungin (ANF) in the WT (B8441), cna1∆ (YSBA99), cnb1∆ (YSBA102), and cnb1∆ cna1∆ (YSBA172) strains. (B) Checkerboard assay results showing the interaction between MIF or ANF and FK506 in the WT strain (B8441). (C) Checkerboard assay results of caspofungin (CAF), MIF, or ANF with cyclosporin A (CsA) in the WT strain (B8441). (TIF) [file ppat.1013363.s006.tif]

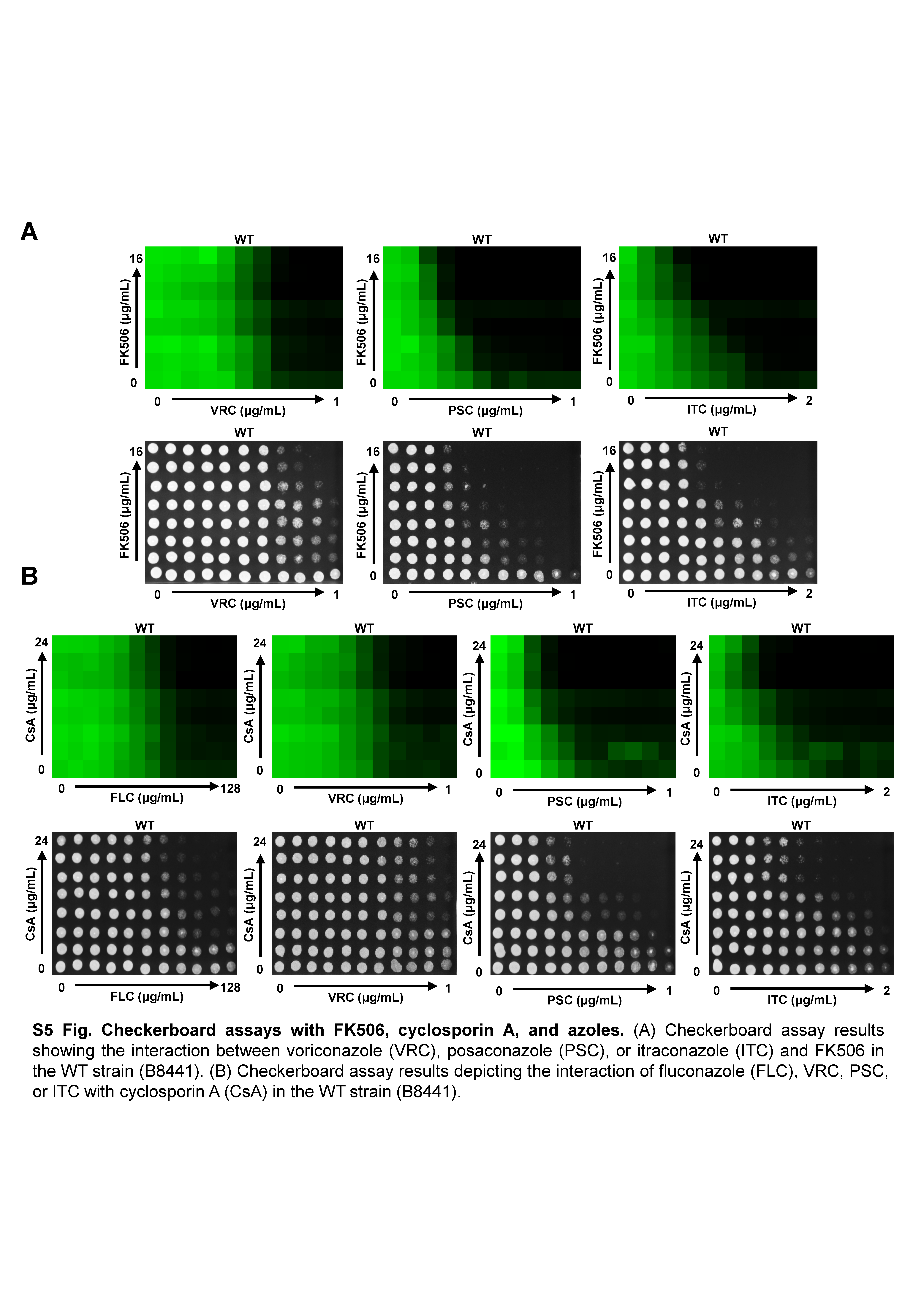

Supplement: S5 Fig — (A) Checkerboard assay results showing the interaction between voriconazole (VRC), posaconazole (PSC), or itraconazole (ITC) and FK506 in the WT strain (B8441). (B) Checkerboard assay results depicting the interaction of fluconazole (FLC), VRC, PSC, or ITC with cyclosporin A (CsA) in the WT strain (B8441). (TIF) [file ppat.1013363.s007.tif]

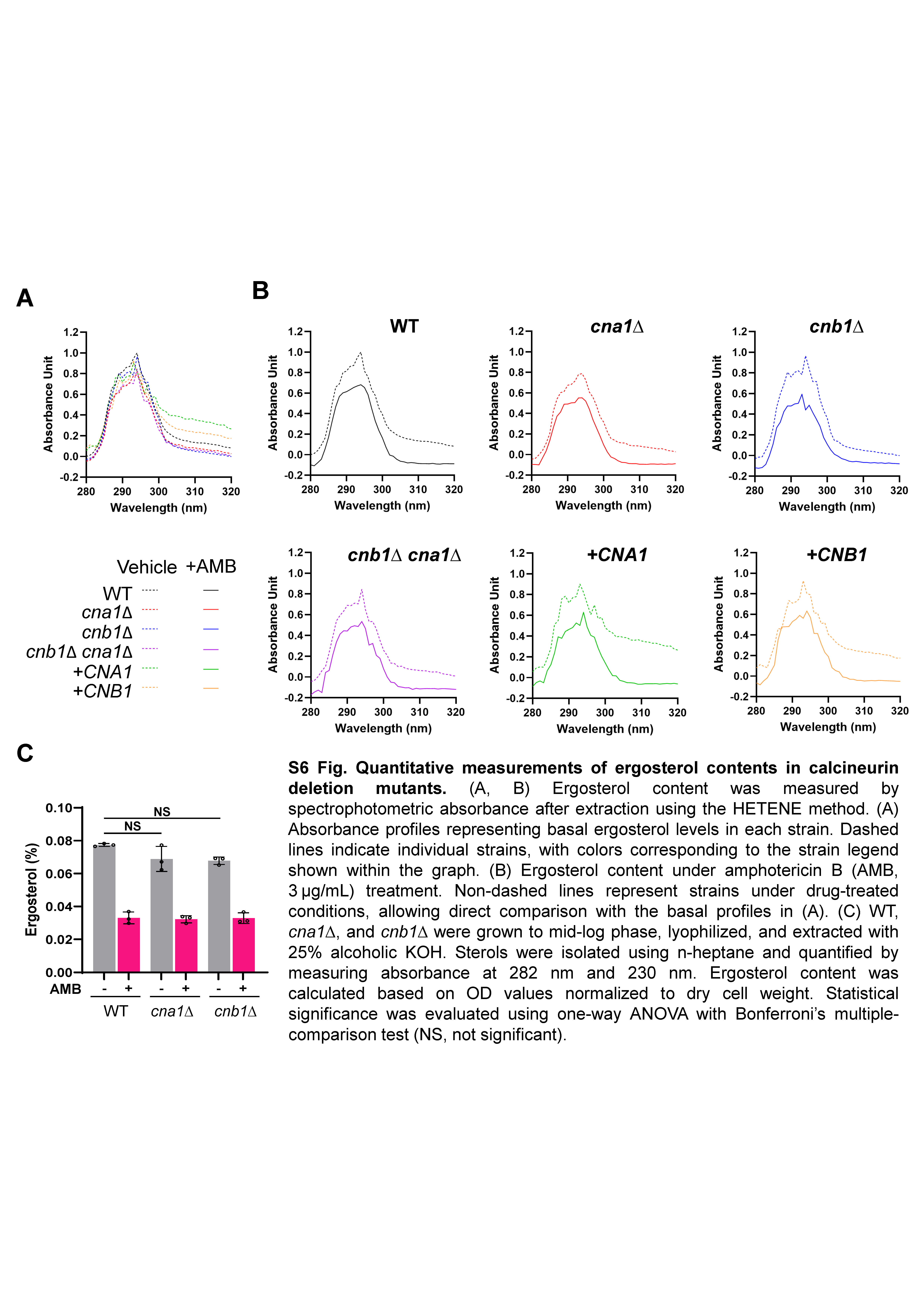

Supplement: S6 Fig — (A, B) Ergosterol content was measured by spectrophotometric absorbance after extraction using the HETENE method. (A) Absorbance profiles representing basal ergosterol levels in each strain. Dashed lines indicate individual strains, with colors corresponding to the strain legend shown within the graph. (B) Ergosterol content under amphotericin B (AMB, 3 μg/mL) treatment. Non-dashed lines represent strains under drug-treated conditions, allowing direct comparison with the basal profiles in (A). (C) WT, cna1∆, and cnb1∆ were grown to mid-log phase, lyophilized, and extracted with 25% alcoholic KOH. Sterols were isolated using n-heptane and quantified by measuring absorbance at 282 nm and 230 nm. Ergosterol content was calculated based on OD values normalized to dry cell weight. Statistical significance was evaluated using one-way ANOVA with Bonferroni’s multiple-comparison test (NS, not significant). (TIF) [file ppat.1013363.s008.tif]

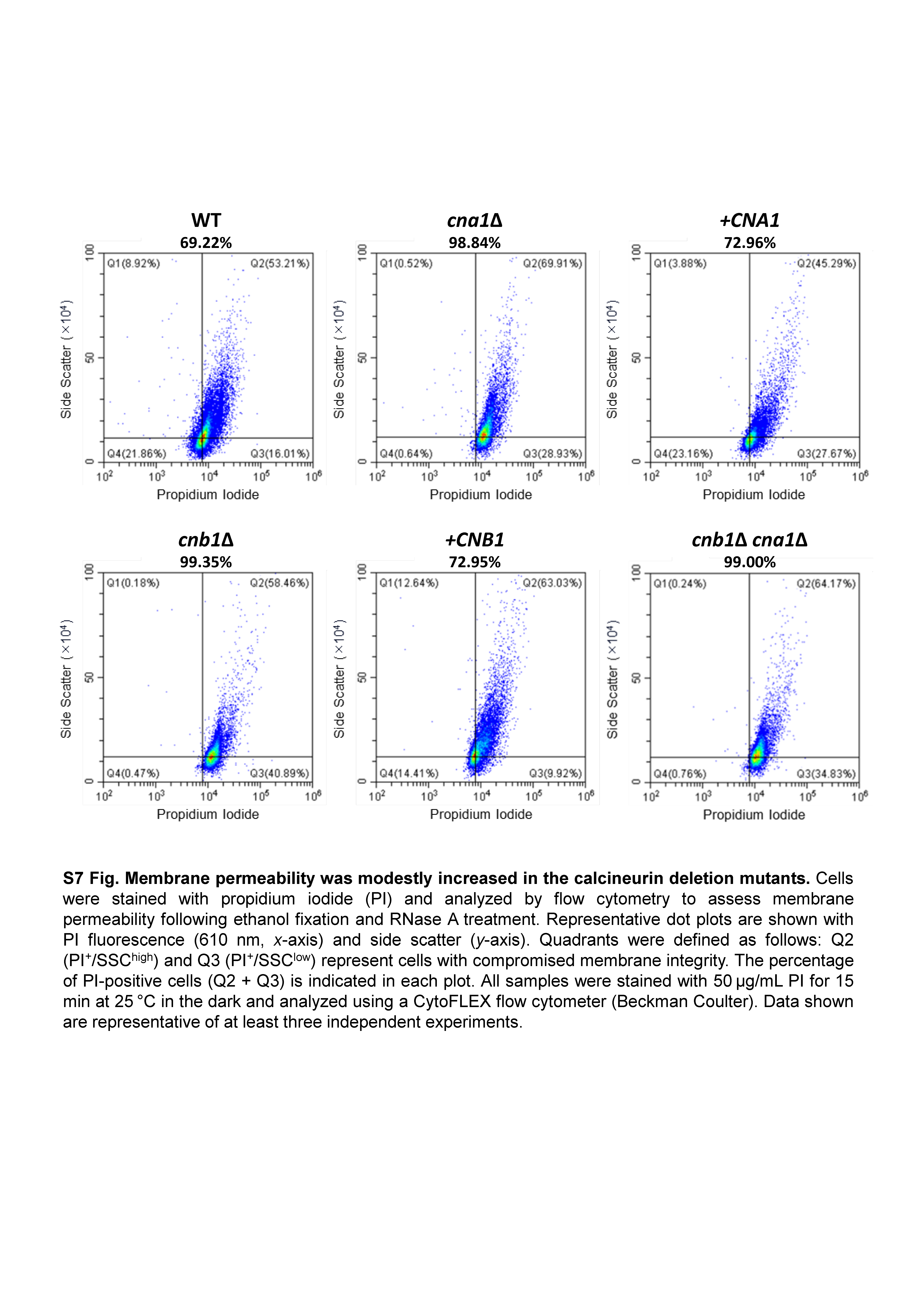

Supplement: S7 Fig — Cells were stained with propidium iodide (PI) and analyzed by flow cytometry to assess membrane permeability following ethanol fixation and RNase A treatment. Representative dot plots are shown with PI fluorescence (610 nm, x-axis) and side scatter (y-axis). Quadrants were defined as follows: Q2 (PI ⁺ /SSChigh) and Q3 (PI ⁺ /SSClow) represent cells with compromised membrane integrity. The percentage of PI-positive cells (Q2 + Q3) is indicated in each plot. All samples were stained with 50 μg/mL PI for 15 min at 25°C in the dark and analyzed using a CytoFLEX flow cytometer (Beckman Coulter). Data shown are representative of at least three independent experiments. (TIF) [file ppat.1013363.s009.tif]

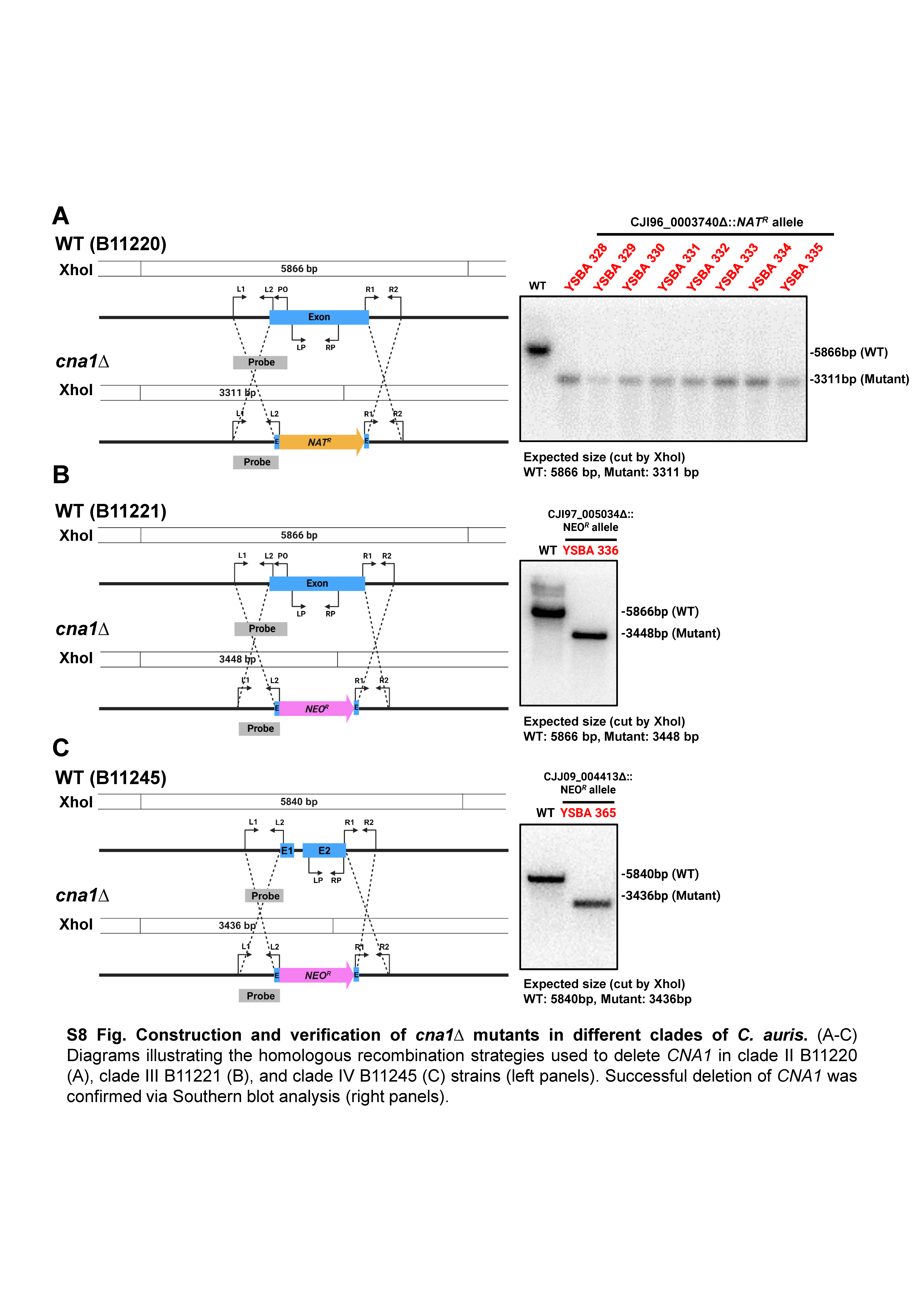

Supplement: S8 Fig — (A-C) Diagrams illustrating the homologous recombination strategies used to delete CNA1 in clade II B11220 (A), clade III B11221 (B), and clade IV B11245 (C) strains (left panels). Successful deletion of CNA1 was confirmed via Southern blot analysis (right panels). (TIF) [file ppat.1013363.s010.tif]

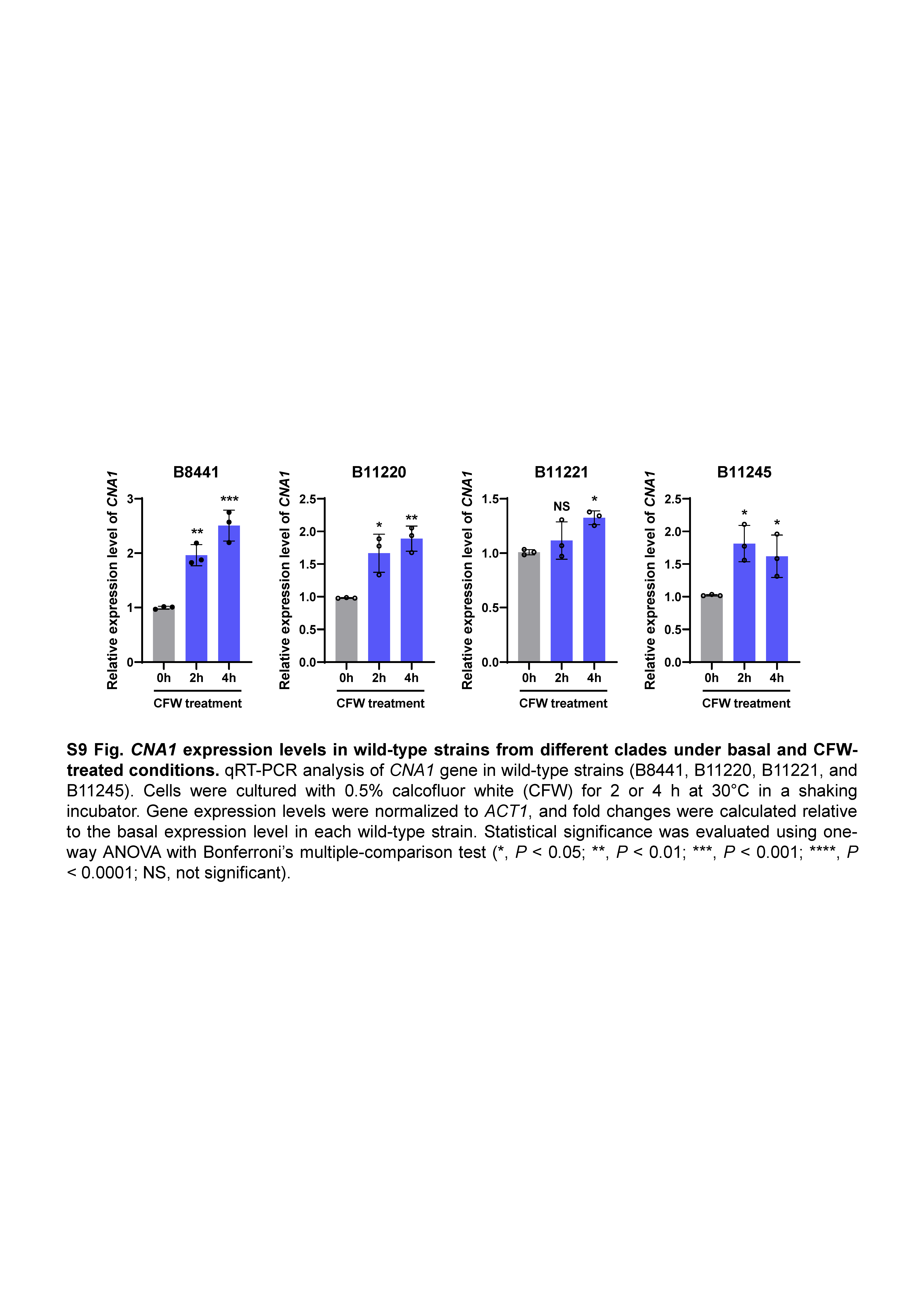

Supplement: S9 Fig — qRT-PCR analysis of CNA1 gene in wild-type strains (B8441, B11220, B11221, and B11245). Cells were cultured with 0.5% calcofluor white (CFW) for 2 or 4 h at 30°C in a shaking incubator. Gene expression levels were normalized to ACT1, and fold changes were calculated relative to the basal expression level in each wild-type strain. Statistical significance was evaluated using one-way ANOVA with Bonferroni’s multiple-comparison test (*, P < 0.05; **, P < 0.01; ***, P < 0.001; ****, P < 0.0001; NS, not significant). (TIF) [file ppat.1013363.s011.tif]

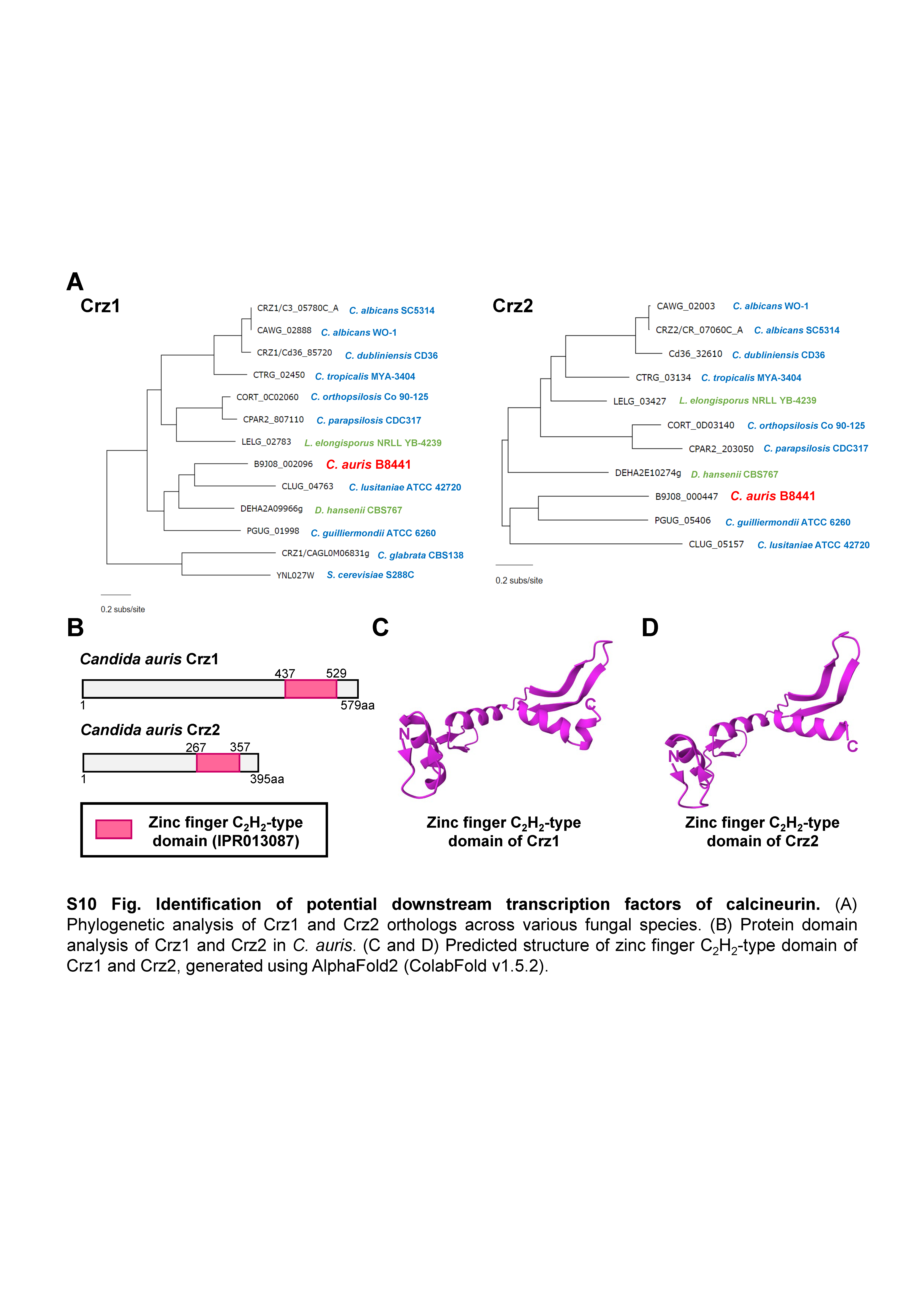

Supplement: S10 Fig — (A) Phylogenetic analysis of Crz1 and Crz2 orthologs across various fungal species. (B) Protein domain analysis of Crz1 and Crz2 in C. auris. (C and D) Predicted structure of zinc finger C2H2-type domain of Crz1 and Crz2, generated using AlphaFold2 (ColabFold v1.5.2). (TIF) [file ppat.1013363.s012.tif]

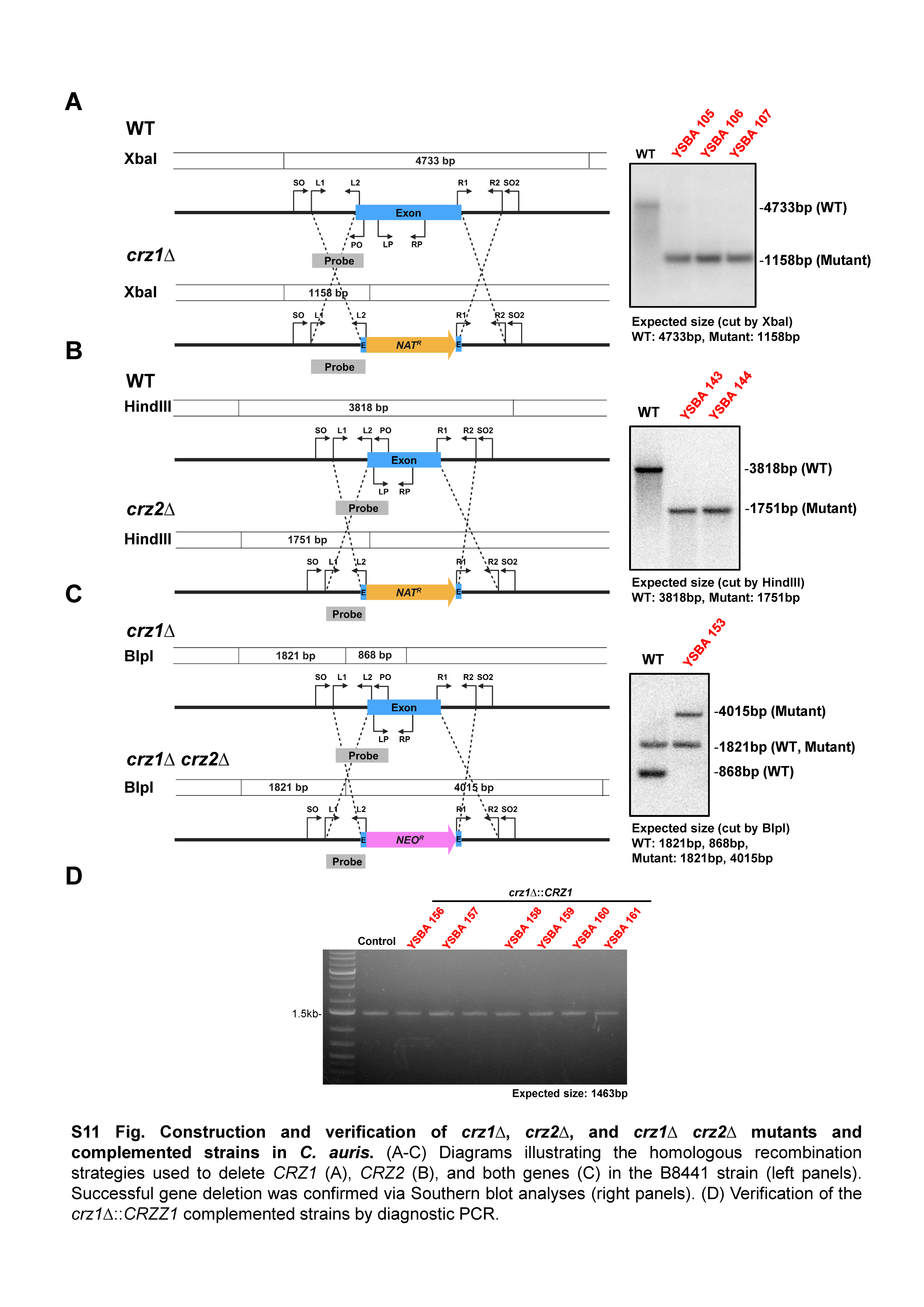

Supplement: S11 Fig — (A-C) Diagrams illustrating the homologous recombination strategies used to delete CRZ1 (A), CRZ2 (B), and both genes (C) in the B8441 strain (left panels). Successful gene deletion was confirmed via Southern blot analyses (right panels). (D) Verification of the crz1∆::CRZZ1 complemented strains by diagnostic PCR. (TIF) [file ppat.1013363.s013.tif]

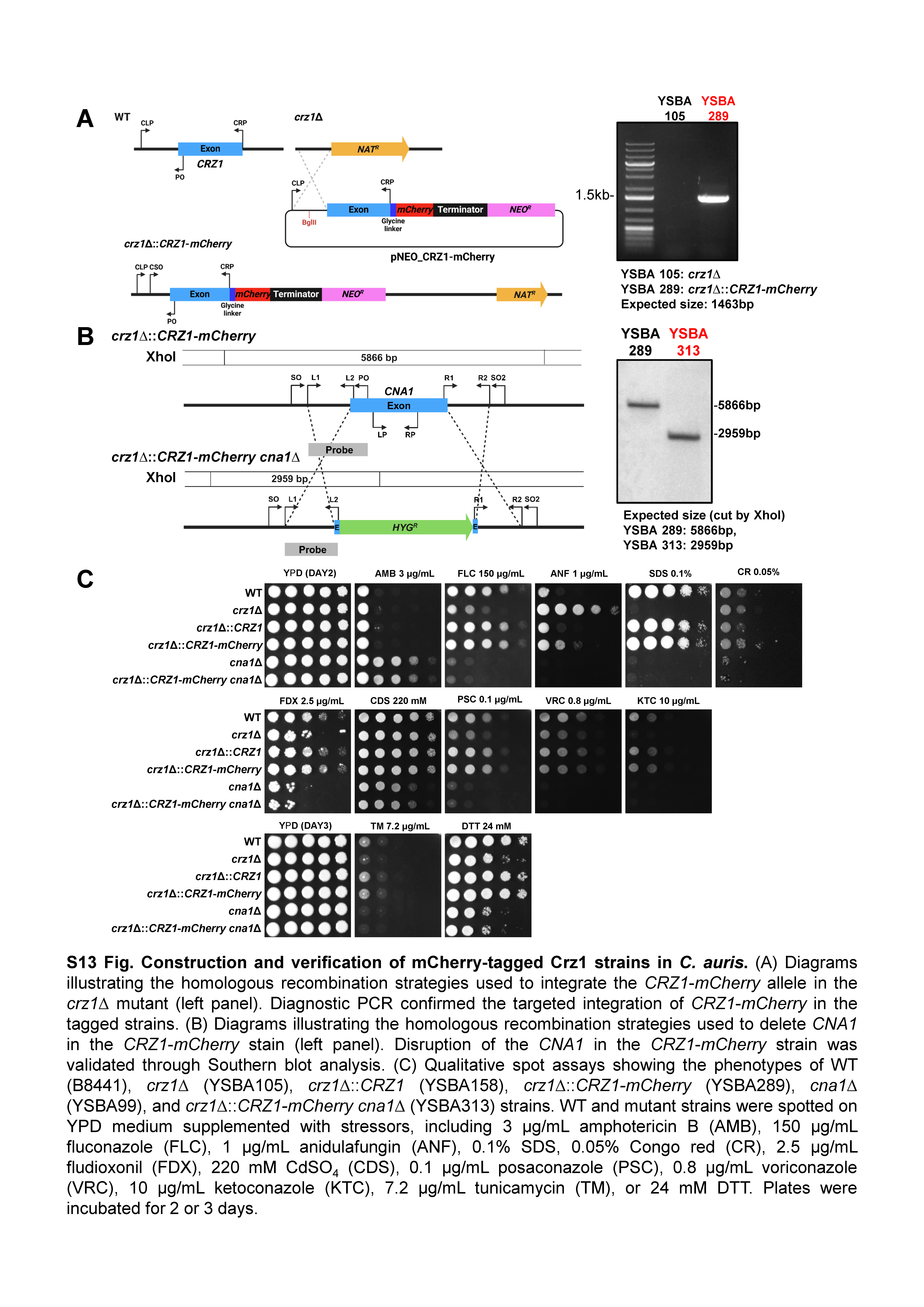

Supplement: S12 Fig — (A) Qualitative spot assays showing the stress susceptibility of WT (B8441), crz1∆ (YSBA105), crz1∆::CRZ1 (YSBA158), crz2∆ (YSBA143), crz1∆ crz2∆ (YSBA153), and cnb1∆ cna1∆ (YSBA172) strains. WT and mutant strains were spotted on YPD medium supplemented with stressors, including 0.5 μg/mL posaconazole (PSC), 0.8 μg/mL voriconazole (VRC), 5 μg/mL ketoconazole (KTC), 0.15 μg/mL micafungin (MIF), or amphotericin B (AMB). Plates were incubated for 2 days. (B) Phenome heat map of each mutant for various stress. Phenotype scores are color-coded based on qualitative or semi-quantitative measurements under the indicated growth conditions. Abbreviations: 30T, 30°C; 37T, 37°C; 42T, 42°C; 45T, 45°C AMB, amphotericin B; FDX, fludioxonil; 5FC, 5-flucytosine; FLC, fluconazole; PSC, posaconazole; ITC, itraconazole; VRC, voriconazole; KTC, ketoconazole; CAF, caspofungin; MIF, micafungin; ANF, anidulafungin; HPX, hydrogen peroxide; TBH, tert-butyl hydroperoxide; DIA, diamide; MD, menadione; MMS, methyl methanesulfonate; HU, hydroxyurea; TM, tunicamycin; DTT, dithiothreitol; CR, Congo red; CFW, calcofluor white; SDS, sodium dodecyl sulfate; CDS, cadmium sulfate; KCR, YPD + KCl; NCR, YPD + NaCl; SBR, YPD + sorbitol; KCS, YP + KCl; NCS, YP + NaCl; SBS, YP + sorbitol. Red and blue gradients represent phenotype reduction and enhancement, respectively, with strong, intermediate and weak phenotypes indicated by color intensity. (TIF) [file ppat.1013363.s014.tif]

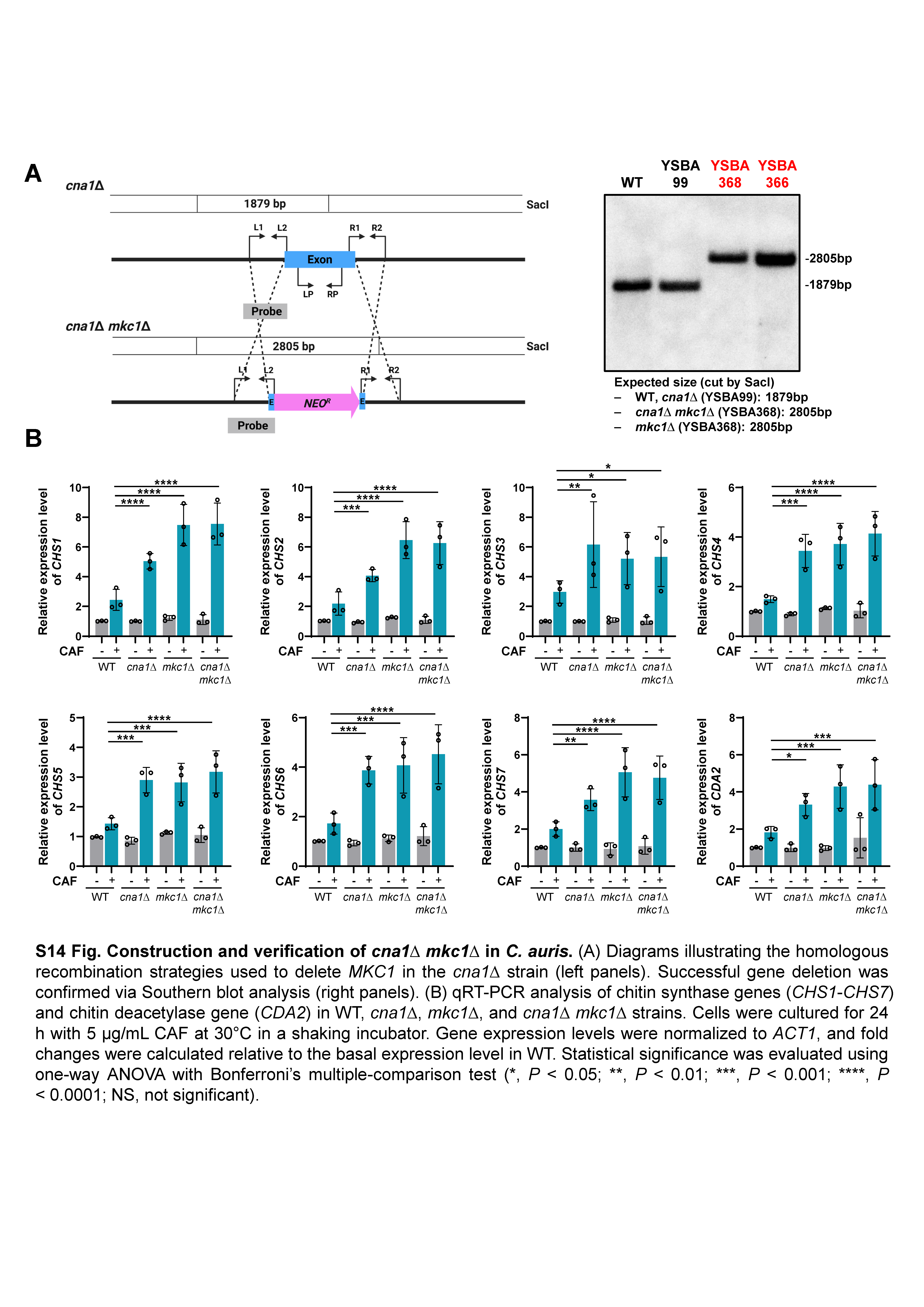

Supplement: S14 Fig — (A) Diagrams illustrating the homologous recombination strategies used to delete MKC1 in the cna1∆ strain (left panels). Successful gene deletion was confirmed via Southern blot analysis (right panels). (B) qRT-PCR analysis of chitin synthase genes (CHS1-CHS7) and chitin deacetylase gene (CDA2) in WT, cna1∆, mkc1∆, and cna1∆ mkc1∆ strains. Cells were cultured for 24 h with 5 μg/mL CAF at 30°C in a shaking incubator. Gene expression levels were normalized to ACT1, and fold changes were calculated relative to the basal expression level in WT. Statistical significance was evaluated using one-way ANOVA with Bonferroni’s multiple-comparison test (*, P < 0.05; **, P < 0.01; ***, P < 0.001; ****, P < 0.0001; NS, not significant). (TIF) [file ppat.1013363.s016.tif]

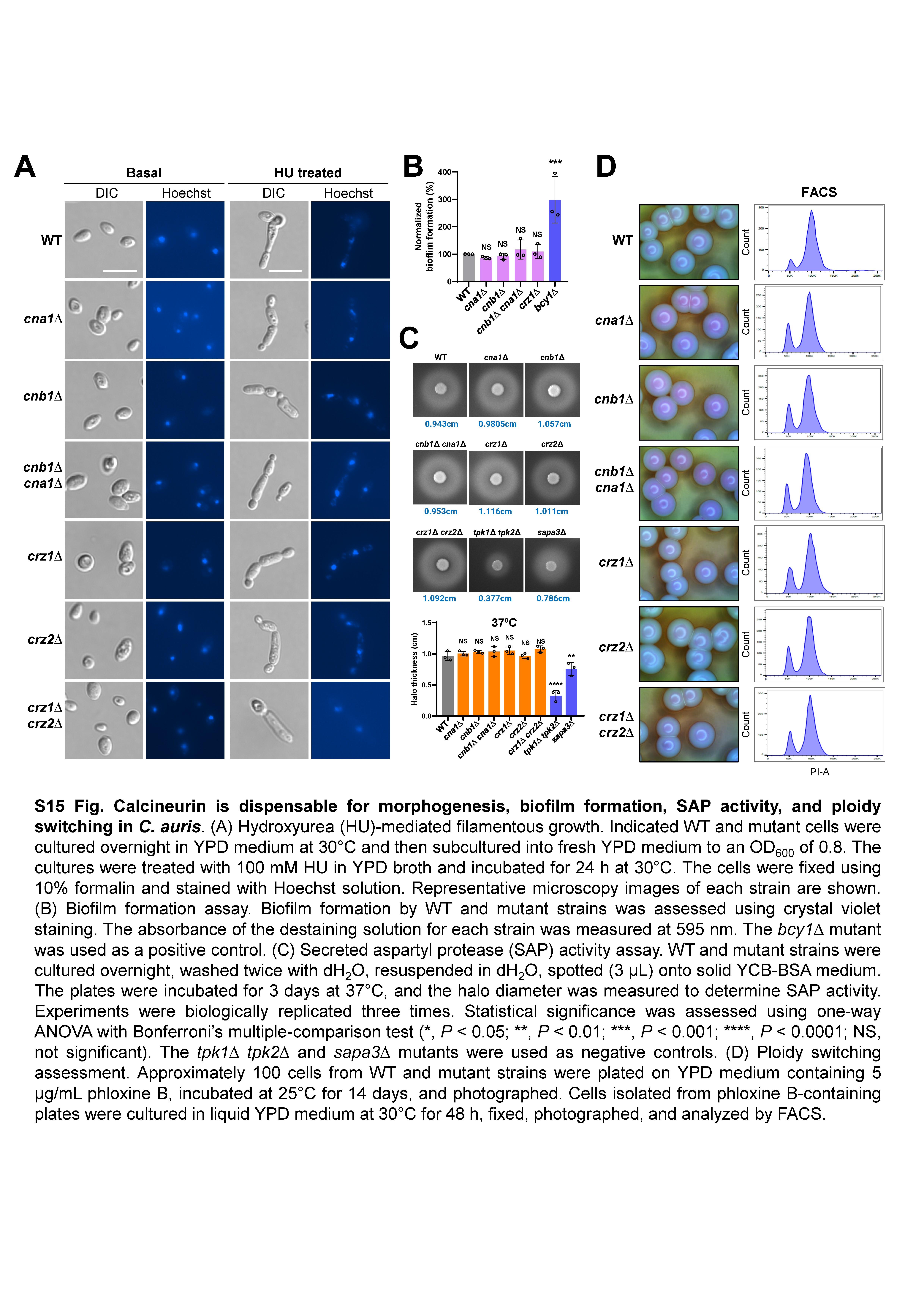

Supplement: S15 Fig — (A) Hydroxyurea (HU)-mediated filamentous growth. Indicated WT and mutant cells were cultured overnight in YPD medium at 30°C and then subcultured into fresh YPD medium to an OD600 of 0.8. The cultures were treated with 100 mM HU in YPD broth and incubated for 24 h at 30°C. The cells were fixed using 10% formalin and stained with Hoechst solution. Representative microscopy images of each strain are shown. (B) Biofilm formation assay. Biofilm formation by WT and mutant strains was assessed using crystal violet staining. The absorbance of the destaining solution for each strain was measured at 595 nm. The bcy1∆ mutant was used as a positive control. (C) Secreted aspartyl protease (SAP) activity assay. WT and mutant strains were cultured overnight, washed twice with dH2O, resuspended in dH2O, spotted (3 μL) onto solid YCB-BSA medium. The plates were incubated for 3 days at 37°C, and the halo diameter was measured to determine SAP activity. Experiments were biologically replicated three times. Statistical significance was assessed using one-way ANOVA with Bonferroni’s multiple-comparison test (*, P < 0.05; **, P < 0.01; ***, P < 0.001; ****, P < 0.0001; NS, not significant). The tpk1∆ tpk2∆ and sapa3∆ mutants were used as negative controls. (D) Ploidy switching assessment. Approximately 100 cells from WT and mutant strains were plated on YPD medium containing 5 μg/mL phloxine B, incubated at 25°C for 14 days, and photographed. Cells isolated from phloxine B-containing plates were cultured in liquid YPD medium at 30°C for 48 h, fixed, photographed, and analyzed by FACS. (TIF) [file ppat.1013363.s017.tif]
