# Supplementary material for: The calcineurin pathway regulates extreme thermotolerance, cell membrane and wall integrity, antifungal resistance, and virulence in Candida auris
Source: PLoS Pathog. 2025 Jul 28;21(7):e1013363. doi: 10.1371/journal.ppat.1013363 (PMC12324677; doi:10.1371/journal.ppat.1013363)
Supplement: Attachment — Submitted filename: [Response to Reviewers] PPATHOGD-25-00022.docx [file ppat.1013363.s019.docx]

**Response to Reviewers**
**Reviewers' Comments:**
Reviewer's Responses to Questions

**Part I - Summary**
Please use this section to discuss strengths/weaknesses of study, novelty/significance, general execution and scholarship.

**Reviewer #1:** This study investigated the role of the calcineurin pathway in the fungal pathogen Candida auris. The CNA1 and CNB1 genes encode the catalytic and regulatory subunits of calcineurin, respectively. The researchers constructed mutants deleted for either gene or both genes. They also generated a mutant deleted for the CRZ1 gene, which encodes a transcription factor previously shown to be activated by calcineurin-mediated dephosphorylation in other organisms. Comprehensive phenotypic analyses of these mutants were conducted to assess their responses to high temperature, various antifungal drugs, and chemicals that damage the cell membrane and cell wall. Additionally, their virulence was evaluated using a fruit fly infection model as well as mouse skin and systemic infection models. The authors conclude that the calcineurin pathway employs distinct regulatory mechanisms to perform divergent roles in regulating cell wall and membrane integrity, antifungal drug resistance, and virulence in C. auris.
This is the first comprehensive investigation of the roles of the calcineurin pathway in C. auris. Relevant mutants were constructed and examined under diverse conditions, including high temperature, all three major classes of antifungal compounds, cell wall toxins, membrane-disrupting agents, and DNA-damaging agents, and assessed for virulence using three infection models. In general, experiments were well executed and controlled. The manuscript was well written with proper conclusions.
**One weakness of this work is its lack of mechanistic depth in addressing some very interesting observations.** For example, the high resistance of the calcineurin mutants to amphotericin B versus their susceptibility to azoles, the distinct roles of calcineurin and its downstream transcription factor CRZ1, and the likely existence of additional regulatory targets of calcineurin remain insufficiently explored. Nevertheless, these findings open exciting opportunities for future research.

**Response:** We thank the reviewer for their thoughtful comments and for recognizing the significance and quality of our work. We fully agree that the study work could have gained greater mechanistic depth through the identification of additional transcription factors downstream of the calcineurin complex beyond Crz1. To address this, we also identified and functionally characterized a Crz1 paralog, Crz2. However, our functional analysis indicated that Crz2 operates independently of the calcineurin pathway. As the reviewer rightly noted, further work is needed to identify novel calcineurin-dependent transcription factors. We plan to pursue this in future studies by performing phosphoproteomics analysis of the calcineurin mutants, followed by functional validation of potential candidate transcription factors. We believe that this line of investigation constitutes a substantial project in its own right and extends beyond the scope of the current study. As the reviewer also kindly acknowledged, we hope this work is recognized as the first comprehensive investigation into the roles of the calcineurin pathway in *C. auris*, laying a foundation for future mechanistic explorations.

**Reviewer #2:** This manuscript presents a carefully conducted, thorough characterization of the roles of calcineurin and the calcineurin-dependent Crz1 transcription factor in the emerging pathogenic yeast Candida auris. Using knock-out mutants for C. auris CNA1 and CNB1 encoding the two subunits of calcineurin and CRZ1, the authors have assessed the role of these genes in sensitivity to a variety of stresses including antifungals, in the expression of virulence traits and in pathogenicity using different animal models of C. auris infection.

Notable results include the demonstration that calcineurin is necessary for survival at temperatures above 42°C; for survival upon cell membrane and cell wall stress; for tolerance to azoles and echinocandins and (surprisingly) sensitivity to amphotericin B; Crz1 acts downstream of calcineurin and yet not all phenotypes associated to calcineurin loss are shared with the Crz1 knock-out mutant suggesting complex interplay between these two components of the calcium signaling pathway and the probable involvement of additional downstream effector(s) of calcineurin in this pathway; the calcineurin pathway is dispensable for important virulence traits of C. auris such a biofilm formation, morphogenesis, protease production and ploidiy shifts; and the calcineurin pathway plays contrasting roles in C. auris pathogenicity.

Many of these results are not unexpected given the knowledge that has been gained on the roles of calcineurin and Crz1 in other pathogenic fungal species such as C. albicans, C. glabrata and Cryptococcus neoformans. Yet, presentation of these results is important as there are variations across fungal species in the roles of calcineurin and Crz1. It is noteworthy that, here, the authors show that there are even variations across the different genetic cluster that structure the C. auris population. Data presented in this manuscript pave the way for future studies aimed in particular at refining our understanding of the calcium signaling pathway in C. auris and identifying additional components that explain the phenotypic differences between the calcineurin and crz1 mutants.

**Response:** We thank the reviewer for highly appreciating the significance and quality of our work. As the reviewer correctly pointed out, the calcineurin pathway appears to play conserved roles in *C. auris* in certain phenotypic processes, such as maintaining cell wall integrity, similar to its roles in other fungal pathogens. At the same time, our findings also highlight notable divergence in calcineurin function across fungal species. In this study, we uncovered several unique features of the calcineurin pathway in *C. auris*, including its opposite roles in antifungal resistance, such as increased susceptibility to fluconazole (FLC) and enhanced resistance to amphotericin B (AMB), as well as the differential contribution of calcineurin and Crz1 to cell wall integrity. These findings emphasize the complexity and context-specific regulation of this critical signaling pathway. We believe our study provides valuable insights that broaden the understanding of calcineurin signaling across fungal pathogens and anticipate that it will serve as a key reference for future investigations. As also noted in our response to the reviewer #1, we plan to identify novel calcineurin-dependent transcription factors in future studies to further elucidate this pathway.

**Reviewer #3:** The manuscript by Cha et al presents data on the role of calcineurin pathway in antifungal drug resistance and viruluence of Candida auris. C. auris is a multi drug resistant human fungal pathogen that presents a serious threat to global human health. C. auris also colonizes human skin which is considered a risk factor for patient to patient transmission, especially in health care settings. Thus, the focus of the present study to identify and characterize fungal factors regulating antifungal drug resistance and skin colonization mechanisms is quite significant. The rationale for this study was built from previous published studies implicating calcineurin pathway in stress response, drug resistance and virulence in other Candida species. The manuscript is very well written. The methodology is sound and in most cases the results justify the conclusions. Overall, the findings of this work are very interesting, and may help better understand the contributions of calcineurin pathway in drug resistance and fungal pathogenesis of C. auris.

**Response:** We thank the reviewer for recognizing the importance and quality of our work.

**Part II – Major Issues: Key Experiments Required for Acceptance**
**Reviewer #1:** The observed significant upregulation of CHS genes in the cna1 and cnb1 mutants, but not in the wild type, in response to caspofungin is intriguing. It is known that disruption of glucan synthesis activates CHS expression via the PKC-MAPK cell wall integrity pathway as a compensatory mechanism. The current findings suggest that calcineurin may play a previously unknown role in negatively regulating this pathway. It would be informative if the authors could block the PKC-MAPK pathway in the cna1 mutant to determine whether caspofungin treatment still activates CHS expression.

**Response:** We appreciate the reviewer’s valuable insights into the potential interplay between calcineurin and PKC-MAPK cell wall integrity pathways in regulating chitin synthesis. In response to the reviewer’s suggestion, we further deleted the *MKC1* MAPK gene (B9J08_002682), a key component of the PKC-Mkc1 MAPK pathway, in the *cna1*∆ mutant and assessed *CHS* gene expression. Notably, *CHS* genes remained significantly upregulated in the double mutant (*cna1*∆ *mkc1*∆) as presented in S14 Fig, indicating that the elevated expression observed in the *cna1*∆ mutant is not positively mediated by the canonical PKC-MAPK pathway. These findings suggest the involvement of additional, PKC-independent signaling mechanisms that are activated in the absence of calcineurin and contribute to the compensatory upregulation of chitin biosynthesis under cell wall stress. This conclusion has been incorporated into the revised manuscript as follows:

**[Line 342-352]** To assess whether the induction of *CHS* genes in the *cna1*Δ mutant upon CAF exposure is mediated by the PKC-dependent cell wall integrity pathway, we generated a *cna1*Δ *mkc1*Δ double mutant by deleting *MKC1* (B9J08_002682), a mitogen-activated protein kinase (MAPK) central to the PKC pathway in *C. albicans* (27), in the *cna1*Δ background. Quantitative gene expression analysis revealed that *CHS* gene upregulation was further enhanced in the *cna1*Δ *mkc1*Δ mutant compared to the *cna1*Δ single mutant following CAF treatment (S14 Fig). These results demonstrate that the increased *CHS* expression in the absence of calcineurin is not positively regulated by the PKC-Mkc1 MAPK cascade. Instead, they implicate the involvement of additional PKC-independent signaling pathways that are activated in response to cell wall stress when calcineurin function is impaired.

**Reviewer #2:**

1) The authors make the interesting observation that inactivation of CNA1 or CNB1 or simultaneous inactivation of the two genes leads to increased tolerance to amphotericin B (l. 180-182). They suggest that this may be explained by the role of calcineurin in regulating the ergosterol biosynthesis pathway. In order to support this hypothesis, they provide evidence that azoles trigger increased expression of ERG genes in a calcineurin-depedent manner (Fig. 2E). While this may explain the increased sensitivity of the calcineurin mutants to azoles, it does not necessarily support that the proposal that calcineurin-dependent regulation of ergosterol biosynthesis is responsible for increased amB tolerance of the calcineurin mutants. Notably, loss of calcineurin does not seem to impair ERG genes expression in the absence of azoles. To support their hypothesis, the authors should quantify ergosterol in the membranes of calcineurin mutants and possibly evidence that increasing ergosterol in the calcineurin mutants restores sensitivity to amB.

**Response:** We appreciate the reviewer’s insightful comments and fully agree with the point raised. As noted, no basal expression changes were observed in *ERG11* and *ERG6*, and *ERG11* was not induced by FLC treatment in the calcineurin mutant strains (Figure 2E). However, the observed AMB resistance, which contrasts with FLC susceptibility, does not appear to be directly linked to the expression of ergosterol biosynthesis genes. To clarify the contribution of ergosterol content to AMB resistance, we quantified ergosterol levels in the calcineurin mutants. Our analysis revealed no significant differences in total ergosterol content between the wild-type and calcineurin-deficient strains under both basal and AMB-treated conditions (S6 Fig). These results suggest that the increased AMB resistance observed in the calcineurin mutants is not due to changes in ergosterol abundance. Instead, our results point to differences in cell wall and membrane structure, rather than ergosterol content, as a potential basis for AMB resistance in these mutants. Further studies will be required to elucidate the precise roles of membrane organization and cell wall architecture in this non-canonical resistance mechanism.

We have added the following sentences to the revised manuscript:

**[Line 201-213]** In contrast, there were no appreciable changes in basal expression of *ERG* genes or in total ergosterol content in the calcineurin mutant strains (Fig 2E and S6 Fig). Moreover, even after AMB treatment, total ergosterol levels in calcineurin-deficient strains remained comparable to those in the wild-type strain (S6 Fig), indicating that altered ergosterol abundance is unlikely to account for the AMB resistance observed.

Interestingly, membrane permeability, which could influence azole susceptibility, was slightly increased in the calcineurin mutants, potentially promoting greater intracellular accumulation of FLC (S7 Fig). In contrast, AMB resistance may instead be attributed to changes in cell wall and membrane structure rather than ergosterol levels. These findings support the conclusion that differential responses to FLC and AMB in the calcineurin mutants are likely driven by distinct cellular mechanisms, including altered membrane permeability and cell wall architecture.

2) Similarly, it is unclear why the authors think that the increased sensitivity of the calcineurin mutants to echinocandins can be explained by the lack of change in FKS1 upregulation upon echinocandin addition and the increased upregulation of FKS2 in the calcineurin mutants. If anything, this should contribute to increased tolerance.

**Response:** We agree with the reviewer that the altered *FKS2* expression following echinocandin treatment does not fully explain the increased echinocandin susceptibility observed in the calcineurin-deficient mutant. We speculate that *FKS2* upregulation in the mutant is likely a compensatory response aimed at restoring cell wall integrity in the absence of calcineurin function. It is likely that the general role of calcineurin in maintaining cell wall integrity contributes to the pronounced echinocandin susceptibility. To avoid any potential misinterpretation of our earlier conclusion, we have revised the manuscript by adding the following sentences:

**[Line 218-****223]** In contrast, *FKS2* expression was markedly upregulated in the *cna1*Δ mutant upon echinocandin treatment (Fig. 2F), probably as a compensatory response aimed at restoring cell wall integrity in the absence of calcineurin function (Fig 2F). The increased susceptibility of the *cna1*Δ mutant to echinocandins is more likely attributable to an inherent defect in maintaining cell wall integrity resulting from the loss of calcineurin function.

3) The observation that the CNA1 mutant behaves differently according to the clade background is interesting. Given that the Cna1 proteins from clade 1, 2 and 3 appear identical (Fig. 3A), it is likely that phenotypic differences between the KO mutants in these three clades is the result of different levels of expression of CNA1 or variations in the calcium signaling pathway. While exploring the second hypothesis is clearly beyond the scope of this study, providing data of CNA1 expression levels in the 4 clades would be interesting.

**Response:** We appreciate the reviewer’s valuable insights into the divergent regulatory mechanisms of the calcineurin pathway across different *C. auris* clades. Despite minor phylogenetic differences in Cna1 among clades, particularly between clade IV and clades I-III, the phenotypic outcomes of the *cna1*Δ mutants were generally consistent across clades, with the exception of the contrasting resistance patterns to calcofluor white (CFW) observed between clades I/II and III/IV. This observation suggests that variation in *CNA1* expression levels is unlikely to explain the clade-specific phenotypes. To confirm this hypothesis and address the reviewer’s suggestion, we measured basal and CFW-induced *CNA1* expression levels in wild-type strains representing each clade, normalized to *ACT1* expression. Following CFW treatment, *CNA1* expression was moderately induced in all clades, with no major differences in expression levels. These results indicate that the phenotypic variation seen in the *cna1*Δ mutants is unlikely to stem from clade-dependent *CNA1* expression differences (S9 Fig). Instead, it may reflect variation in downstream effectors of the calcineurin pathway or clade-specific genetic modifiers.

We have added the following sentences to the revised manuscript:

**[Line 242-249]** To investigate whether the phenotypic variation observed among *cna1*∆ mutants could be attributed to differences in *CNA1* expression, we measured both basal and CFW-induced *CNA1* transcript levels in wild-type strains from each clade. Upon CFW treatment, *CNA1* expression was moderately upregulated across all clades, with no substantial differences observed in either basal or CFW-induced levels (S9 Fig). These results suggest that the clade-specific phenotypes are unlikely to result from differential *CNA1* expression, but rather may reflect variation in downstream components or modulators of the calcineurin pathway.

**Reviewer #3:** no major issues with the manuscript

**Response:** We appreciate it.

**Part III – Minor Issues: Editorial and Data Presentation Modifications**

**Reviewer #1:** The cna1 and cnb1 mutants’ significantly susceptibility to azoles and high resistance to amphotericin B is both interesting and puzzling. Many azole-resistant fungal strains exhibit cross-resistance to amphotericin B as both classes of drugs target ergosterol. This deserves more discussion. Does the crz1 mutant also show heightened resistance to amphotericin B?

**Response:** We appreciate the reviewer’s comments. Indeed, opposite susceptibility patterns to FLC and AMB are frequently observed in fungal mutants with defects in *ERG11* regulation. In support of this, our previous systematic analysis of *Cryptococcus neoformans* transcription factors (TFs) identified several mutants lacking TFs potentially involved in *ERG11* regulation that exhibited opposite responses to FLC and AMB (Jung et al. *Nat Commun* 2015). For example, the *sre1*Δ mutant, where *SRE1* encodes the central sterol-regulating TF, exhibits increased resistance to AMB but heightened sensitivity to FLC. This pattern is mechanistically explained by the distinct modes of action of these antifungals: FLC inhibits the enzymatic activity of Erg11, while AMB binds directly to ergosterol, forming lethal pores in the fungal membrane. Thus, increased *ERG11* expression can confer FLC resistance (by increasing enzyme abundance), as can be often seen in FLC-resistant mutants with increased chromosomal copy containing the *ERG11* locus, but also leads to higher ergosterol levels in the cell membrane, enhancing AMB binding and susceptibility. For this reason, we examined *ERG11* expression in *C. auris* calcineurin mutants and also quantified ergosterol content, as suggested by Reviewer #2’. Our measurements showed no significant difference in total ergosterol levels between calcineurin mutants and wild-type strains under both basal and AMB-treated conditions (S6 Fig). Additionally, we apologize for initially omitting information on AMB resistance in the *crz1*Δ mutant; this has now been included in S12 Fig.

To further clarify this point, we have added the following text to the manuscript:

**[Line 561-581]** Interestingly, the *cna1*Δ and *cnb1*Δ mutants exhibited increased resistance to AMB while showing heightened susceptibility to azoles. This seemingly paradoxical pattern has been observed in other fungal species and reflects the fundamentally distinct mechanisms of action of these two antifungal drug classes. Azoles inhibit Erg11 enzymatic activity, thereby blocking ergosterol biosynthesis, while AMB binds directly to ergosterol in the membrane, forming lethal pores. As previously reported in *C. neoformans*, disruption of transcription factors regulating *ERG11* expression often leads to opposite susceptibility to FLC and AMB. For instance, *sre1*Δ mutants, where *SRE1* encodes the central sterol-regulating TF (44), exhibit increased resistance to AMB but greater sensitivity to FLC (45). In line with this, *ERG11* expression was moderately increased in the calcineurin mutants following FLC treatment, which may contribute to FLC susceptibility. However, ergosterol quantification showed no significant differences between wild-type and calcineurin mutant strains, suggesting that ergosterol abundance does not account for the observed AMB resistance. To elucidate the underlying mechanisms by which calcineurin deletion confers AMB resistance, transcriptome analyses of wild-type and *cna1*Δ strains under both untreated and AMB-treated conditions will be necessary. These analyses will enable the identification of calcineurin-regulated gene networks and downstream effectors implicated in AMB resistance, potentially uncovering novel pathways involved in cell wall and membrane remodeling, stress adaptation, and antifungal tolerance that compensate for the loss of calcineurin signaling.

Lines 67-69. The description of calcineurin activation is not clear. I thought that the simultaneous binding of Ca/calmodulin to Cna1 and calcium to Cnb1 leads to calcineurin activation, as you correctly show in Fig 7.

**Response:** We appreciate the reviewer’s comment. To clarify the mechanism of calcineurin activation, we revised the sentences as follows:

**[Lines 64-68]** The influx of Ca^2+^ is sensed by calmodulin, leading to the formation of the Ca^2+^-calmodulin complex that binds to Cna1. Concurrently, cytosolic Ca^2+^ also binds to Cnb1, inducing a conformational change in Cna1 that facilitates its interaction with the Ca^2+^/calmodulin complex, thereby activating calcineurin (4).

Lines 74-75, in yeast

**Response:** Corrected.

Lines 174. The observed fungicidal activity is a combined effect of FK506 or cyclosporin A with echinocandins. Thus, it is not correct to say that ‘both FK506 and cyclosporin A demonstrated fungicidal antifungal activity.’ Also, in the description from line 173 to 179, echinocandin concentrations used should be mentioned.

**Response:** We appreciate the reviewer’s comment. In response, we have revised the manuscript to improve clarity regarding the synergistic antifungal activity and to specify the concentrations of echinocandins used as follows:

**[Line 173-175]** However, FK506 and CsA exhibited fungicidal activity only when combined with echinocandins, suggesting a synergistic effect.

**[Line 175-177]** The drug combinations were tested using the following echinocandin concentrations: CAF (up to 20 μg/mL), MIF (1 μg/mL), and ANF (1 μg/mL).

Line 190, The result of ERG6 expression shown in Fig 2E should be described here. It is not mentioned anywhere in the text.

**Response:** We appreciate the reviewer’s comment. In response, we have added the following sentence to clarify differential regulation of ergosterol biosynthetic genes:

**[Line 199-201]** By contrast, *ERG6* expression was induced in the calcineurin mutants upon FLC treatment, suggesting that not all ergosterol biosynthetic genes are regulated by the calcineurin pathway.

Lines 197-199, How does the significantly increased expression of *FKS2* in the cna1 mutant explain its high susceptibility to echinocandins? Shouldn’t the increased expression of a drug target increase resistance to the drug? Need more discussion.

**Response:** This comment reiterates Reviewer 2’s second major point, which we have addressed in detail in our previous response. Please refer to our response and the revised text added to lines 218-223 for comprehensive explanation.

Lines 265-266, The subtitle is confusing. Did you mean to say ‘Crz1 and calcineurin play distinct roles in resistance to cell wall-damaging stressors and echinocandins in C. auris.’?

**Response:** We agree with the reviewer’s comment and have revised the subtitle accordingly:

**[Line 304-305]** Crz1 and calcineurin play distinct roles in resistance to cell wall-damaging stressors and echinocandins in *C. auris*.

**Reviewer #2:**

- l. 50: the figure drawn from reference 1 has been debated, maybe replace “over” by “an estimated” or provide a range from drawn from different studies.

**Response:** We appreciate the reviewer’s comment and have revised the sentence as follows:

**[Line 48-50]** Fungal infections pose a significant global health threat, accounting for an estimated 3.75 million deaths annually, with nearly 1 million of these attributable to *Candida* bloodstream infections or invasive candidiasis (1).

- l. 53-55: “often”, “high mortality rates”: it would be preferable to provide figures, ranges for these statements

**Response:** We agree with the reviewer’s comment. We revised the sentence as follows:

**[Line 50-53]** The fungal pathogen *Candida auris* has emerged as a major clinical concern due to its ability to infect immunocompromised patients and its rapid spread in healthcare settings, with reported mortality rates approaching 34% (2).

- l. 68: revise “altering its conformation of Cna1”

**Response:** We agree with the reviewer’s comment. We revised the sentence as follows:

**[Line 66-68]** Concurrently, cytosolic Ca^2+^ also binds to Cnb1, inducing a conformational change in Cna1 that facilitates its interaction with the Ca^2+^/calmodulin complex, thereby activating calcineurin (4).

- l. 102: provide a reference for CGD

**Response:** As suggested, we have added the website address for the *Candida* genome database (http://www.candidagenome.org/).

- l. 111-112: How robust is the Alphafold3 prediction for these two proteins and the complex they form relative to existing 3D structures? Add a reference for Alphafold3.

**Response:** We thank the reviewer for this insightful question. According to the recent *Nature* publication by the AlphaFold developers (Baek et al., 2024, *Nature*), AlphaFold3 exhibits substantially improved accuracy compared to previous versions—not only in predicting individual protein structures but also in modeling protein–protein complexes. The model achieves near-experimental accuracy across a wide range of targets, with an average TM-score exceeding 0.9 when benchmarked against experimentally resolved structures. Notably, for heterodimeric complexes, AlphaFold3 achieves interface RMSD values within ~2 Å in the majority of test cases, indicating a high degree of structural reliability. We have now added the reference for AlphaFold3 in the revised manuscript to support the structural predictions in Line 112.

- l. 121: add references

**Response:** We added references in Line 122.

- l; 128: here or in the discussion, the authors might want to discuss the relevance of high temperatures (>42°C) in C. auris lifestyle…

**Response:** Thank you for the insightful comment. We have now added a brief discussion addressing the relevance of high-temperature tolerance (>42°C) in the lifestyle and pathogenicity of *Candida auris*. Specifically, we highlighted that *C. auris* displays remarkable thermotolerance compared to other *Candida* species, which may contribute to its persistence in healthcare environments, colonization of human skin, and potential survival in febrile hosts. The following sentence has been added the Discussion section:

**[Line 464-471]** The thermotolerance of *C. auris*, particularly its ability to survive and grow at temperatures ≥42°C, may play a pivotal role in its environmental persistence and transmission. As global temperatures rise due to climate change, new ecological niches with elevated temperatures are emerging. The ability of *C. auris* to survive in such warmer environments may enhance its ability to colonize new habitats, increasing the likelihood of interaction with human hosts and facilitating its spread. This exceptional ability to adapt to and persist in high-temperature conditions may have been a key factor in its emergence as a globally distributed pathogen.

- l. 148: it would probably be more appropriate to use tolerance instead of resistance as the ability of C. auris to grow at the studied concentrations of antifungals is an intrinsic property of the species rather than a genetically-acquired change in phenotype.

**Response:** We appreciate the reviewer’s comment and agree with the distinction. We believe the term “resistance” is appropriate only in cases where a substantial increase in MIC is observed by inheritable genetic mutations, such as the AMB resistance seen in the calcineurin-deficient mutants and echinocandin resistance observed in the *crz1*Δ mutant. Therefore, we have used the term “tolerance” to describe the intrinsic property of the wild-type *C. auris*.

- l. 164-165: Fig. 2B is a representation of the EUCAST test for MIC. It does not provide evidence that azoles become fungicidal in the absence of calcineurin. To demonstrate this, the authors have added one step that is described in the methods section. This should be indicated in order to support the statement (the same comment applies to the statement l. 178).

**Response:** We appreciate the reviewer’s comment. We have included the growth assessment data, as described in the Methods section, in Figure 2B and S4A Fig to provide direct evidence supporting the fungicidal effect of drugs in the calcineurin mutants.

- l. 177: why aren’t the actual FIC values given in the text?

**Response:** We agree with the reviewer’s comment. The actual FIC values have now been added to lines 177-186 as follows:

**[Line 177-186]** Assuming an MIC of 16 μg/mL for FK506 and 24 μg/mL for CsA, the fractional inhibitory concentration (FIC) indices for their combinations with echinocandins were as follows: FK506 combined with CAF, MIF, and ANF yielded FIC indices of <0.03, 0.14, and <0.08, respectively. Similarly, CsA combined with CAF, MIF, and ANF yielded FIC indices of <0.13, 0.14, and <0.1. All FIC indices were consistently below 0.5 (Fig 2C and S4B-C Fig), indicating strong synergistic interactions. Additionally, FK506 and CsA exhibited synergistic fungicidal antifungal activity when combined with azoles. The FIC indices were <0.08 (FK506 + FLC), <0.08 (CsA + FLC), <0.03 (FK506 + PSC), <0.04 (CsA + PSC), <0.15 (FK506 + VRC), <0.16 (CsA + VRC), 0.05 (FK506 + ITC), and 0.06 (CsA + ITC) (Fig 2D and S5 Fig).

- l. 227: evolutionary

**Response:** Corrected

- l. 229: it is surprising that the authors do not report the sensitivity of the crz1 mutant to extreme temperatures

**Response:** We thank the reviewer for the valuable suggestion. In response, we conducted additional experiments to assess the temperature sensitivity of the *crz1*∆ mutant. Both qualitative and quantitative growth data have been added to Figure 4. The corresponding revised text is now included in Lines 270-277 as follows:

**[Line 270-277]** The *crz1*Δ mutant displayed no growth defects at temperatures up to 42°C, similar to the calcineurin mutants (Fig 4A and C). However, at temperatures exceeding 42°C, the *crz1*Δ mutant exhibited impaired growth, suggesting that Crz1, like calcineurin, contributes to extreme thermotolerance (Fig 4B and C). Notably, the growth defect in the *crz1*Δ mutant was less pronounced than that observed in the calcineurin mutants (Fig 4B and C). These findings suggest that while Crz1 plays a supporting role in extreme thermotolerance, calcineurin appears to have a broader and more critical function.

- l. 271: why “still”

**Response:** Corrected.

- l. 332: should read bcy1

**Response:** Corrected.

- l. 343-345: the interest of using a subcutaneous model of infection is unclear given the physiopathology of C. auris. one would argue that skin colonization rather than subcutaneous infection should be preferentially studied.

**Response:** We appreciate the reviewer’s comment regarding the relevance of a subcutaneous infection model to *C. auris* physiopathology. To address this concern, we developed an ex vivo skin attachment assay using murine ears, which more closely mimics surface colonization and enables assessment of the initial interaction between *C. auris* and host skin tissue. This model better reflects its clinical behavior of *C. auris* as a colonizing pathogen. Notably, the *cna1*Δ mutant exhibited a clear defect in early skin colonization, suggesting a role for calcineurin in mediating host surface attachment. These findings have been incorporated in the revised manuscript results section and Figure 6E.

**[Line 399-406]** Next, an ex vivo skin adhesion model was utilized to evaluate the ability of *C. auris* to attach to mammalian skin tissue, a critical step in its colonization and infection process. Adhesion of each strain to murine ear skin tissue was quantified, and the *cna1*Δ mutant showed significantly reduced adhesion at the early 1-h time point compared to the wild-type and complemented strains. However, no significant difference was observed after 2 h of incubation (Fig. 6E). These results suggest that calcineurin signaling is particularly important during the early stage of *C. auris* tissue attachment.

**Reviewer #3:** The authors report very interesting data with azole, echinocandin and polyene susceptibity / resistance. As noted by the authors, it is quite intriguing opposite phenotypes of drug susceptibility of cna and cnb mutants with azoles and amphotericin B. The authors hypothesize that these changes may be due to increased membrane permeability of cna or cnb mutants. Thus, it may be of interest to perform either rhodamine 6g or fluorescein diacetate (FDA) uptake assay with these mutants to demonstrate membrane permeability changes in these strains.

**Response:** We appreciate the reviewer’s valuable insights into the potential role of the calcineurin pathway in regulating membrane permeability. In response, we conducted membrane permeabilization assays using propidium iodide (PI) staining followed by flow cytometric analysis in the calcineurin deletion mutants. These assays revealed a modest but reproducible increase in PI uptake in the mutants compared to the wild-type strain, indicating enhanced membrane permeability. This may contribute to the increased to FLC observed in the calcineurin mutants (S7 Fig). However, since AMB primarily targets ergosterol and exerts its antifungal activity by binding directly to the fungal membrane, without requiring intracellular entry, the observed increase in membrane permeability is unlikely to explain the AMB resistance phenotype. Instead, additional factors such as altered cell wall and membrane architecture may collectively contribute to this resistance. Please also refer to our previous response to Reviewer 1’s first minor comment.
